# Disruption of *Morrbid* alleviates autoinflammatory osteomyelitis in *Pstpip2*-deficient mice

**DOI:** 10.1242/dmm.052176

**Published:** 2025-07-07

**Authors:** Qingran Huo, Jiayu Ding, Hongxi Zhou, Yue Wang, Shanshan Wang, Hang He, Lorie Chen Cai, Jingjing Liu, Ge Dong, Zhigang Cai

**Affiliations:** ^1^ Tianjin Key Laboratory of Inflammatory Biology, Department of Pharmacology, School of Basic Medical Science, Tianjin Medical University, Tianjin 300070, China; ^2^ State Key Laboratory of Experimental Hematology, Tianjin Medical University, Tianjin 300070, China; ^3^ The Province and Ministry Co-sponsored Collaborative Innovation Center for Medical Epigenetics, School of Basic Medical Science, Tianjin Medical University, Tianjin 300070, China; ^4^ Department of Bioinformatics, School of Basic Medical Science, Tianjin Medical University, Tianjin 300070, China; ^5^ Department of Hematology, Tianjin Medical University Tianjin General Hospital, Tianjin 300070, China; ^6^ Department of Rheumatology and Immunology, Tianjin Medical University Tianjin General Hospital, Tianjin 300070, China

**Keywords:** Autoinflammatory diseases, Chronic osteomyelitis, *Pstpip2*, *Morrbid*, Osteoclasts, Single-cell RNA sequencing, Bone damage

## Abstract

Autoinflammatory diseases (AIDs) are defined as abnormal activation of the innate immune system leading to spontaneous and uncontrolled inflammation. AIDs may affect bone tissue and lead to chronic recurrent multifocal osteomyelitis (CRMO). However, the etiology and treatment of CRMO remain elusive. In previous studies, we reported that loss of *Morrbid* prevents myeloid-lineage leukemogenesis. Here, we observed that *Morrbid* and *Pstpip2* are co-expressed in mature myeloid cells and hypothesize a pathogenic role for *Morrbid* in osteomyelitis. We generated a *Pstpip2^−/−^* strain with a 5-bp deletion in *Pstpip2*, and the strain manifests CRMO-like phenotypes*.* Loss of *Morrbid* in *Pstpip2^−/−^* mice significantly inhibited the initiation and progression of CRMO symptoms and mitigated activation of myeloid cells and the excessive release of inflammatory cytokines. In addition, single-cell transcriptome analysis demonstrated reduction of osteoclasts and inflammatory cells caused by loss of *Morrbid* in the *Pstpip2^−/−^Morrbid^−/−^* compound mutants. Using murine models, this study profiles the pathological cell landscape of CRMO by single-cell analysis and suggests that reducing the lifespan of inflammatory myeloid cells by targeting *Morrbid* can be an effective therapy for chronic osteomyelitis.

## Teaser

**Summary:** Using single-cell RNA-sequencing analysis and knockout of *Morrbid* in *Pstpip2^−/−^* mice, we show that reducing the lifespan of inflammatory myeloid cells by targeting *Morrbid* can be an effective therapy for chronic osteomyelitis.

## INTRODUCTION

Inflammatory diseases caused by dysfunction of innate immune cells are called autoinflammatory diseases (AIDs) (Krainer et al., 2020; Szekanecz et al., 2021). At present, many types of AIDs have been identified in clinics, including colitis, dermatosis, chondritis, osteomyelitis, vasculitis and VEXAS disease (a recently reported rheumatoid-hematological syndrome) (Meier-Schiesser and French, 2021; Rood and Behrens, 2022; Beck et al., 2020). However, treatments for AIDs are limited (Rood and Behrens, 2022; Georgin-Lavialle et al., 2019). More insightful understanding of the pathogenesis of AIDs is therefore needed to develop more accurate and effective treatments.

Chronic recurrent multifocal osteomyelitis (CRMO) is a non-bacterial autoinflammatory bone disease characterized by multifocal and multiple recurrences in a progressive pattern (Stern and Ferguson, 2013). The typical symptom of CRMO is pain at the bony injury site with or without swelling, which tends to occur around the metaphysis of the femur, tibia and clavicle (Stern and Ferguson, 2013; Cox and Ferguson, 2018). Patients with CRMO are frequently affected by other chronic inflammatory diseases or autoimmune diseases, including arthritis, psoriasis and inflammatory bowel disease, suggesting that systematic inflammation is affected in the disease (Stern and Ferguson, 2013; Cox et al., 2017). Recently, a variant in *IL1R1* (p.Lys131Glu) was reported to cause CRMO in a juvenile female with profound inflammatory myeloid cells in peripheral blood (Wang et al., 2023), indicating the involvement of IL-1β signaling and circulating inflammatory cells in CRMO. However, only the peripheral mononuclear blood cells (PBMCs) of the patient were profiled by single-cell RNA sequencing (scRNA-seq) in the *IL1R1* variant-related study. We argue that profiling the immune and stromal cells in the affected region is necessary to provide more adequate pathological information and to assist with understanding the pathogenesis of CRMO.

A mouse model *Pstpip2^cmo/cmo^* (with a missense point-mutation in *Pstpip2*; ‘*cmo*’, chronic multifocal osteomyelitis) shows pathological characteristics reminiscent of human CRMO and has been widely used as an animal model for pathophysiological dissection of CRMO and general AIDs (Byrd et al., 1991; Ferguson et al., 2006). *Pstpip2^cmo/cmo^* mutant mice spontaneously develop CRMO starting at the age of 2 months, typically characterized by progressive hind paw swelling and deformity, skin and ear inflammation, and inflammatory bone resorption. It is also suggested that such inflammation is mediated by myeloid-skewed hematopoiesis and inflammatory myeloid cells (such as neutrophils and macrophages) of the innate immune system (Chitu et al., 2012; Cassel et al., 2014; Yao et al., 2022; Kralova et al., 2020).

As an adaptor protein residing on the cell membrane, proline-serine-threonine-phosphatase-interacting protein 2 (PSTPIP2) is reported to play a role in regulating membrane curvature and maintaining cytoskeleton stability by binding to its interaction partners [typically phosphatases, i.e. PTPN12 and SHIP1 (also known as INPP5D)]. PSTPIP2 is functionally correlated with the migration and activation of macrophages and osteoclasts (a highly specified macrophage for bone-absorbing) (Xu et al., 2021; Kralova et al., 2021). Previous studies indicate that inflammasome, caspases and reactive oxygen species (ROS) all are involved in the pathological development in *Pstpip2^cmo/cmo^* mice (Kralova et al., 2020; Lukens et al., 2014). Interestingly, mutations in *PSTPIP1* result in PSTPIP1-associated myeloid-related proteinemia inflammatory syndrome, a rare AID (Zhang et al., 2021). However, to our knowledge, no disorder indications have been reported with mutations in *PSTPIP2* in humans. Nonetheless, mechanisms by which PSTPIP2 regulates myeloid cells, especially the phagocytotic cells (i.e. osteoclasts, macrophages and neutrophils), have not been fully elucidated, and the cell populations, cytokines and signaling pathways involved in the occurrence and cascade of CRMO for *Pstpip2^cmo/cmo^* mice are also controversial. Furthermore, effective interventions or drugs to inhibit the progression of CRMO remain to be explored.

Our previous studies have demonstrated that a conserved long non-coding RNA (lncRNA), *Morrbid*, is specifically expressed in mature eosinophils, neutrophils and classical monocytes and promotes myeloid-lineage leukemogenesis (Kotzin et al., 2016; Cai et al., 2018, 2021, 2020a,b). In addition, *Morrbid* was reported to have an intimate connection with the release of various cytokines and activity of cell signaling pathways (Cai et al., 2018, 2021; Kotzin et al., 2019; Zohar et al., 2023). This prompted us to examine whether *Morrbid* (also known as *MIR4435-2HG* in human) could be targeted to inhibit the progression of AIDs including CRMO.

In addition, scRNA-seq technologies provide new assets as powerful as the traditional approaches for dissecting the pathogenesis of immune diseases. It is believed that single-cell omics are revolutionizing the study of immunology and rheumatology (Baslan and Hicks, 2017). Single-cell sequencing technologies can provide a systematic map (a cellular atlas) for affected tissues and their microenvironment alteration. As mentioned above, even though a PBMC profile has been described in an *IL1R1*-variant-carrying patient with CRMO (Wang et al., 2023), so far, extensive understanding of the affected tissues in humans or even in mice is still lacking.

In the present study, we showed that *Pstpip2* and *Morrbid* are co-expressed in myeloid cells. We generated a new mouse strain with a frameshift mutation (5-bp deletion) in *Pstpip2* (referred to as *Pstpip2^−/−^* hereafter) and validated that its phenotypes are similar to those in the classical *Pstpip2^cmo/cmo^* strain. We generated double-knockout (DKO) mutants, *Pstpip2^−/−^Morrbid^−/−^* to investigate the involvement of *Morrbid* in osteomyelitis. Deficiency of *Morrbid* in the DKO mice inhibited the progression of inflammatory symptoms and bone damage, mediated by the decrease in myeloid cells and cytokines in the lesion site. The mitigation of CRMO symptoms in the DKO mice was also validated by scRNA-seq and flow cytometry analyses using the affected bone marrow (BM) cells from *Pstpip2^−/−^* and DKO strains.

## RESULTS

### A 5-bp deletion in Exon 2 of *Pstpip2* leads to CRMO

**Fig. 1.**
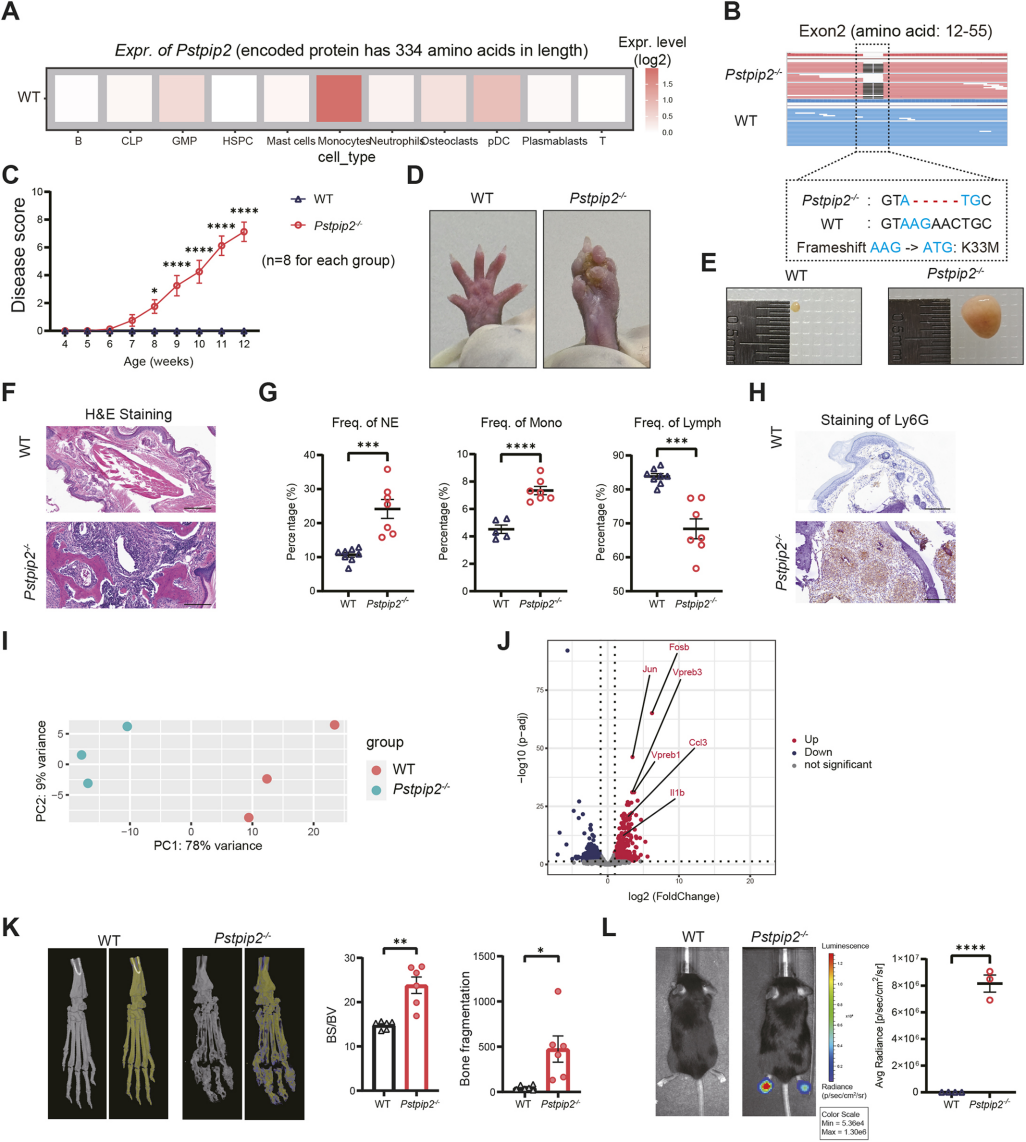
**A 5-bp deletion in *Pstpip2* leads to chronic osteomyelitis in mice**. (A) Expression levels of *Pstpip2* in various compartments of wild-type (WT) bone marrow (BM) cells, revealed by single-cell RNA sequencing (scRNA-seq). See also Fig. 3 for details. B, B cells; CLP, common lymphoid precursors; GMP, granulocyte-monocyte progenitors; HSPC, hematopoietic stem and progenitor cells; pDC, plasmacytoid dendritic cells. (B) Generation of *Pstpip2^−/−^* mice. The 5-bp deletion is in the coding region, in Exon 2 of the gene locus. The range of amino acids encoded by Exon 2 is 12-55, and the full length of the Pstpip2 protein is 334 amino acids. The upper panel shows the transcripts revealed by the bulk RNA sequencing (RNA-seq) analysis; the lower panel shows the Sanger sequencing results. The frameshift mutation caused by the 5-bp deletion in *Pstpip2^−/−^* mice is highlighted. The frameshift of AAG to ATG (in blue) results in K33M miscoding at the first and all the downstream frameshift mutations. (C) Disease progression was scored (scale from 0 to 10) by visual inspection of the hind paws of WT and *Pstpip2^−/−^* mice. *n*=8 per group; two-way ANOVA, mean±s.e.m. (D,E) Representative images of the hind paws (D) and popliteal lymph nodes (E) of 20-week-old mice. *n*=3 per group. (F) Representative Hematoxylin and Eosin (H&E) staining images of hind paw sections (scale bars: 200 μm). *n*=3 per group. (G) Frequencies of neutrophils (NE), monocytes (Mono) and lymphocytes (Lymph) in the peripheral blood (*n*≥5 per group). (H) Representative immunohistochemical (IHC) images of Ly6G on hind paw sections. Stain in brown, neutrophil marker Ly6G (scale bars: 200 μm). (I) Principal component (PC) analysis of WT and *Pstpip2^−/−^* mice by bulk RNA-seq. (J) Volcano plots of differentially expressed genes (DEGs) in BM cells of WT and *Pstpip2^−/−^* mice by bulk RNA-seq. *n*=3 per group. (K) Representative micro-computed tomography (micro-CT) of hind paw bones from 20-week-old mice. Gray images show total bone tissue, while pseudo-color images provide differences between high-density (yellow) and low-density (blue) areas. Quantifications of bone surface (BS)/bone volume (BV) and bone fragmentation are shown on the right. *n*=6 per group. (L) Measurement and quantification of superoxide in WT and *Pstpip2^−/−^* mice. The mice were injected [intraperitoneally (i.p.)] with L-012 chemiluminescent probe before imaging. If not stated otherwise, unpaired two-tailed Student's *t*-test was performed; statistical results are presented as mean±s.e.m.; *P<0.05, **P<0.01, ***P<0.001, ****P<0.0001.

**Fig. 2.**
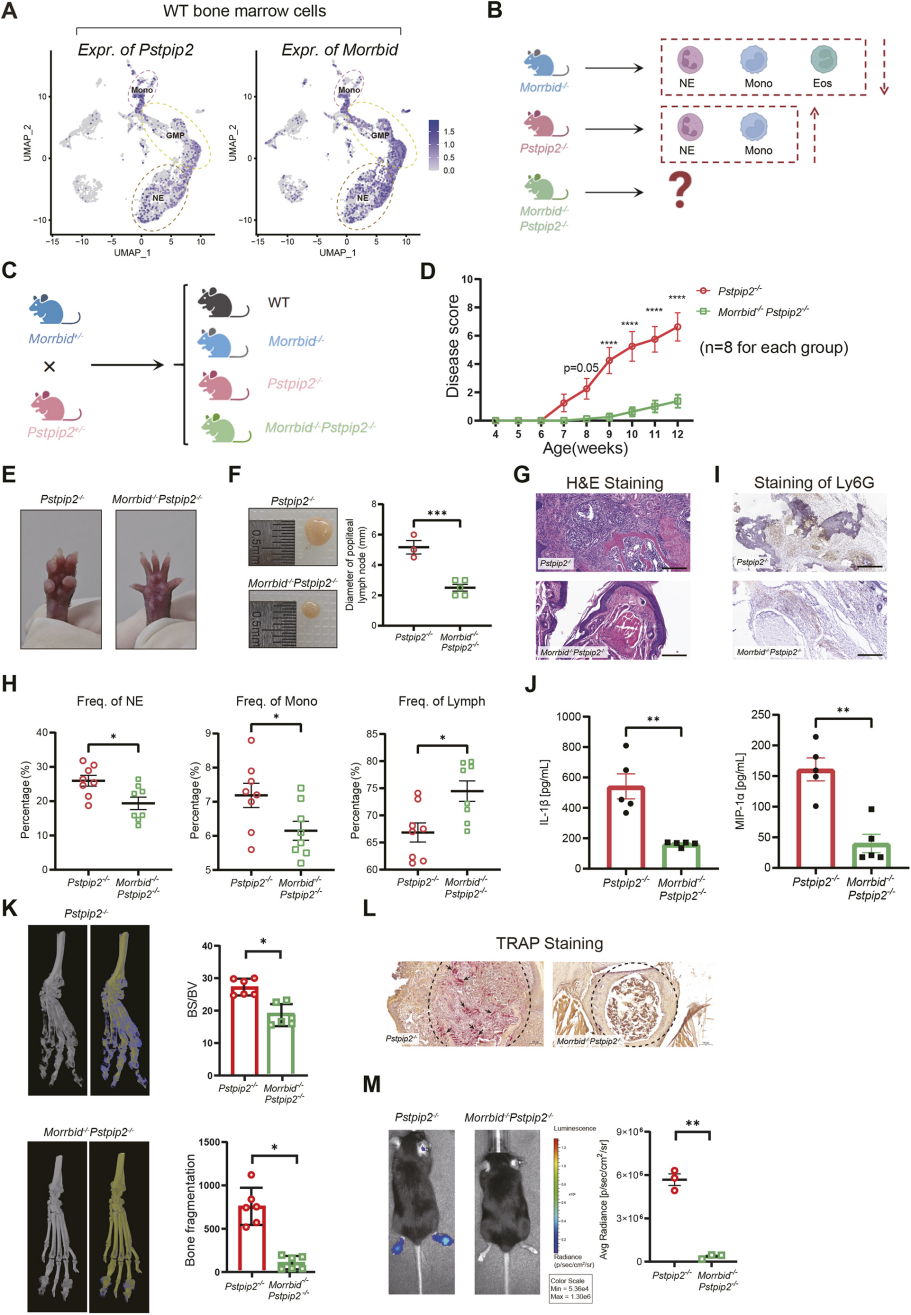
**Loss of *Morrbid* significantly inhibits chronic recurrent multifocal osteomyelitis (CRMO) progression in *Pstpip2^−/−^* mice.** (A) Expression of *Pstpip2* and *Morrbid* in the scRNA-seq dataset of BM cells from WT hind paws. GMP, granulocyte-macrophage-progenitors; Mono, monocytes; NE, neutrophils. (B) Schematic for the rationale and hypothesis for this study. Red dashed line boxes indicate three major myeloid cell types. Compared to WT mice, *Pstpip2^−/−^* mice have greater numbers of myeloid cells whereas *Morrbid^−/−^* mice have fewer myeloid cells. Eos, eosinophils. (C) Schematic of the generation of the compound mutant mice: the *Pstpip2^−/−^Morrbid^−/−^* double-knockout (DKO) mice. (D) Disease severity was scored (scale from 0 to 10) by visual inspection of the hind paws. *n*=8 per group; two-way ANOVA, mean±s.e.m. (E) Representative images of the hind paws of *Pstpip2^−/−^* and *Pstpip2^−/−^Morrbid^−/−^* mice at the age of 20 weeks. (F) Representative images and quantification (size in diameter) of the popliteal lymph nodes. *n*=3 for *Pstpip2^−/−^*, *n*=4 for *Pstpip2^−/−^Morrbid^−/−^*. Age, 20 weeks. (G) Representative H&E images of hind paw sections of *Pstpip2^−/−^* and *Pstpip2^−/−^Morrbid^−/−^* mice (scale bars: 200 μm). *n*=3 per group. (H) Frequencies of NE, Mono and Lymph in the peripheral blood of *Pstpip2^−/−^* and *Pstpip2^−/−^Morrbid^−/−^* mice. *n*=8 per group. (I) Representative IHC images of hind paw sections of *Pstpip2^−/−^* and *Pstpip2^−/−^Morrbid^−/−^* mice. Stain in brown, neutrophil marker Ly6G (scale bars: 200 μm). *n*=3 per group. (J) IL-1β and MIP-1α (CCL3) concentration detected by enzyme-linked immunosorbent assay. *n*=5 per group; tissues, hind paw lysates. (K) Representative micro-CT of hind paw bones from 20-week-old *Pstpip2^−/−^* and *Pstpip2^−/−^Morrbid^−/−^* mice. *n*=6 per group. Quantifications of BS/BV (top) and bone fragmentation (bottom) are shown on the right. (L) Representative histological sections of hind paws stained by TRAP (a marker for osteoclasts; arrows); the dashed line marks the BM cavity region (scale bars: 100 μm). *n*=3 per group. (M) Measurement and quantification of superoxide. The mice were injected (i.p.) with L-012 chemiluminescent probe before imaging. Unpaired two-tailed Student's *t*-test, mean±s.e.m.; *P<0.05, **P<0.01, ***P<0.001, ****P<0.0001.

Using the scRNA-seq dataset, we confirmed that *Pstpip2* was highly expressed in murine myeloid-lineage cells, especially monocytes in wild type (WT) (Fig. 1A; see also Fig. 2A). We constructed a mouse strain carrying a frameshift mutation (referred to as *Pstpip2^−/−^*) by knocking out 5 bp in the *Pstpip2* protein-coding region [Exon 2, the frameshift of AAG to ATG (blue in Fig. 1B) results in K33M miscoding and all downstream frameshift mutations; full-length Pstpip2 protein has 334 amino acids]. The mice manifest obvious osteomyelitis, and the main lesion is in the bilateral hind paws of mice. We continuously observed the hind paws of both WT and *Pstpip2^−/−^* mice over the age of 2-6 months and rigorously documented the occurrence and progression of the disease. We established a scoring system: each swollen toe counts as 1 point, with a total score of 10 points as the end point of the disease. Through continuous observation and scoring, we found that the hind paw swelling of *Pstpip2^−/−^* mice was characterized by progressive aggravation (Fig. 1C). Almost all *Pstpip2^−/−^* mice showed marked swelling in all toes of bilateral hind paws by the age of 12 weeks (penetration rate is 100%; Fig. 1C), and their appearance was dramatically different from that of WT mice (Fig. 1D). We isolated popliteal lymph nodes (popLNs) from 20-week-old WT and *Pstpip2^−/−^* mice and observed massive lymphomegaly of bilateral popLNs in *Pstpip2^−/−^* mice, suggesting inflammatory symptoms in nearby tissues (Fig. 1E). These results confirm that the newly generated *Pstpip2^−/−^* mice show a spontaneous, non-bacterial inflammatory disease, reminiscent of the classic *Pstpip2^cmo/cmo^* phenotype.

**Fig. 3.**
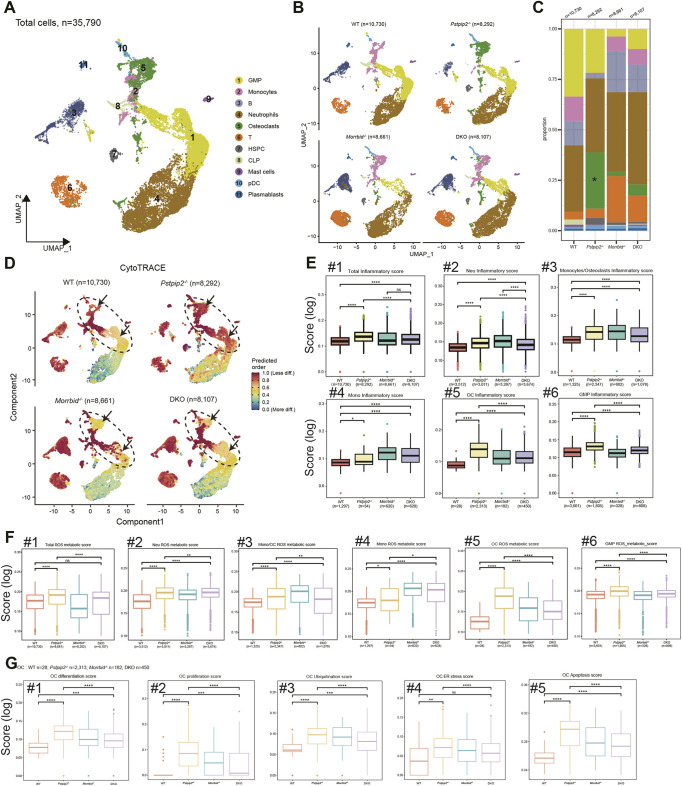
**scRNA-seq analysis of the BM cells from the hind paws of four genotypes of mice.** (A) Uniform manifold approximation and projection (UMAP) visualization of the BM cells (*n*=35,790); each dot represents a cell, and each color indicates a cell type as indicated. T, T cells. (B) Separate UMAP plots of BM cells from the four genotypes of the mice. (C) Stacked chart of the average proportion of each cell type in the four genotypes of the mice. (D) Heatmap for the stemness scores of BM cells from the four genotypes of mice. The computing tool CytoTRACE was utilized to analysis cell stemness. Greater values (dark red) suggest a state with greater stemness [less differentiated and more primitive (progenitor cell-like)]. Note that osteoclasts, neutrophils and GMPs from *Pstpip2^−/−^* mice appear more immature compared with those from the other three genotypes. (E,F) Scoring of various gene signatures as indicated in the four genotypes of mice. (E) The total level and individual types of inflammatory gene signature scores in myeloid cells as indicated. Neu, neutrophils; OC, osteoclasts. (F) Additional scoring analysis focusing on reactive oxygen species (ROS) metabolic gene signature scores of the BM cells. (G) Various aspects of scoring in the OC compartments. Utilizing the scRNA-seq datasets, we also measured the activity of OCs by computing (1) differentiation score, (2) proliferation score, (3) ubiquitination score, (4) endoplasmic reticulum (ER) stress score and (5) apoptosis score of OCs from the four genotypes of mice. Unpaired two-tailed Student's *t*-test. mean±s.e.m.; ns, not significant; *P<0.05, **P<0.01, ***P<0.001, ****P<0.0001.

*Pstpip2^−/−^* mice had very high levels of immune cells and granulocyte infiltration in the hind paws, whereas WT mice showed no such infiltration (Fig. 1F). Accordingly, in the peripheral blood of *Pstpip2^−/−^* mice, we found that the frequency of neutrophils and monocytes was significantly increased, whereas the frequency of lymphocytes was inversely decreased (Fig. 1G). The results of immunohistochemical (IHC) staining showed that *Pstpip2^−/−^* mice had more abundant neutrophils in the hind paw tissue compared to WT (Fig. 1H). These results suggest that myeloid cells, including neutrophils and monocytes, were likely the major cell types that drive osteomyelitis caused by *Pstpip2* deficiency.

We then performed bulk RNA sequencing (RNA-seq) on BM cells from WT and *Pstpip2^−/−^* mice to compare differences at the transcriptome level (Fig. 1I). Analysis of differentially expressed genes (DEGs) showed that *Pstpip2^−/−^* mice had significantly increased mRNA expression levels of cytokines, including *Il1b* and *Ccl3*, compared to WT (Fig. 1J). Studies have shown that cytokines such as IL-1β and CCL3 (also known as MIP-1α) act as osteoclast-activating factors and promote the dissolution of bone matrix in a variety of diseases (Yokota et al., 2021; Yao et al., 2021; Mori et al., 2011; Roodman, 2001, 2006; Toh et al., 2004). In addition, micro-computed tomography (micro-CT) results showed that there were multiple low-density areas in the hind paw bone tissue of *Pstpip2^−/−^* mice, compared with that of WT mice, suggesting severe damage to the bone tissue (Fig. 1K). Phagocytes (especially neutrophils) are central to the inflammatory response, and nicotinamide adenine dinucleotide phosphate (NADPH) oxidase present in them is one of the major sources of ROS (Griffith et al., 2009; Mittal et al., 2014). ROS are not only involved in regulating cell growth, differentiation, apoptosis and activation of cell signaling cascades as intracellular second messengers, but also mediate inflammation by oxidizing protein and lipid cellular components and damaging DNA (Mittal et al., 2014; Agidigbi and Kim, 2019). Moreover, ROS are also important components in regulating osteoclast differentiation and activation (Agidigbi and Kim, 2019). We therefore visualized ROS production in living mice using the luminol derivative L-012. We observed a more intense luminescence signal in the hind paws of *Pstpip2^−/−^* mice compared with that in the hind paws of WT mice (Fig. 1L), suggesting that ROS overproduction is involved in the genesis of inflammation and osteolysis.

### Deletion of *Morrbid* inhibits CRMO-like phenotypes in *Pstpip2^−/−^* mice

By regulating its downstream pro-apoptotic gene *Bcl2l11 in cis*, *Morrbid* regulates apoptosis and controls the lifespan of mature myeloid cells, such as eosinophils, neutrophils and monocytes in blood and tissues (Kotzin et al., 2016). We therefore investigated the expression levels of *Morrbid* and *Pstpip2* in murine myeloid cells. Using scRNA-seq, we observed that both *Morrbid* and *Pstpip2* were specifically and highly expressed in myeloid cells such as monocytes, granulocyte-monocyte progenitors (GMPs) and neutrophils (Fig. 2A).

Given the massive expansion of myeloid cells in the peripheral blood and hind paw sites of the *Pstpip2^−/−^* mice, we hypothesized that *Morrbid* deletion slows the progression of CRMO-like symptoms (Fig. 2B). DKO mice (*Pstpip2^−/−^Morrbid^−/−^*) were generated by crossing *Morrbid^+/−^* mice with *Pstpip2^+/−^* mice. *Pstpip2^−/−^* mice were used as controls for further study of the function of *Morrbid* in osteomyelitis (Fig. 2C). We found that *Morrbid* knockout significantly attenuated and delayed the development of hind paw swelling caused by *Pstpip2* depletion using our scoring system (Fig. 2D). Furthermore, the effect was durable rather than temporary, as evidenced by our observations in the hind paws of two group mice at the age of 20 weeks (Fig. 2E). We also found that *Pstpip2^−/−^Morrbid^−/−^* mice had apparently smaller popliteal lymph nodes than those of *Pstpip2^−/−^* mice, suggesting that inflammation in the surrounding tissues was diminished (Fig. 2F).

We next addressed the effects of *Morrbid* on immune cells in *Pstpip2^−/−^* mice. Hematoxylin and Eosin (H&E) staining of the hind paws of mice showed that *Pstpip2^−/−^Morrbid^−/−^* mice had significantly reduced infiltration of immune cells and inflammatory granulomas (Fig. 2G). Hemogram analysis of peripheral blood from the two groups of mice showed that the *Pstpip2^−/−^Morrbid^−/−^* mice had a significantly reduced frequency of neutrophils and monocytes, and an increased frequency of lymphocytes, compared with the *Pstpip2^−/−^* mice (Fig. 2H). IHC staining of the hind paws revealed a significant reduction in the number of neutrophils in *Pstpip2^−/−^Morrbid^−/−^* mice (Fig. 2I). Excessive activation of immune cells releases a mass of cytokines and enhances the self-feedforward of inflammation (Jarczak and Nierhaus, 2022). Hind paw homogenates of the two groups of mice were obtained for enzyme-linked immunosorbent assay (ELISA) with the aim of measuring the protein levels of IL-1β and MIP-1α (CCL3) in hind paws. The results showed significantly lower levels of the two cytokines in hind paws from the *Pstpip2^−/−^Morrbid^−/−^* mice than in those from the *Pstpip2^−/−^* mice (Fig. 2J).

In addition, we quantified bone damage in the DKO mice. By micro-CT, we confirmed that knockout of *Morrbid* in *Pstpip2^−/−^* mice resulted in an evident reduction in low-density areas and a significantly decreased degree of osteolysis (Fig. 2K). Bone tissue homeostasis is synergistically maintained as supported by osteoblastic bone formation and osteoclastic bone resorption (Ono and Nakashima, 2018). Tartrate-resistant acid phosphatase (TRAP; also known as ACP5), a specific marker of osteoclasts, plays a critical role in the synthesis and degradation of bone tissue (Hayman, 2008; Ballanti et al., 1997; Halling Linder et al., 2017; Harada et al., 2013). By staining TRAP in hind paw sections from both groups of mice, we found that *Pstpip2^−/−^Morrbid^−/−^* mice had fewer osteoclasts and, accordingly, an increased complete BM cavity in the hind paw (Fig. 2L). These results suggest that the rescue of bone damage in *Pstpip2^−/−^* mice by *Morrbid* deletion is likely due to inhibition of osteoclast formation. We also evaluated the effect of *Morrbid* deletion on ROS production, observing a significant reduction in ROS luminescence signal in *Pstpip2^−/−^Morrbid^−/−^* mice compared with that in *Pstpip2^−/−^* mice after intraperitoneal injection of the ROS reporter L-012 (Fig. 2M). In conclusion, the deletion of *Morrbid* significantly reduced the frequencies of myeloid cells and the levels of inflammatory cytokines in the hind paws of CRMO mice.

### scRNA-seq reveals CRMO pathogenesis in *Pstpip2^−/−^* mice and confirms *Morrbid* loss-mediated interventions in DKO mice

scRNA-seq analysis was performed on the hind paws of four groups of mice (WT, *Morrbid^−/−^*, *Pstpip2^−/−^* and DKO). Muscle cells were removed, and only cells from the BM were included for the library construction and sequencing procedure (see Materials and Methods). After the Seurat annotation pipeline and filtering mesenchymal cells, ∼8000-10,000 hematopoietic (CD45^+^; also known as PTPRC^+^) cells per group were included for further annotation analysis. Annotation of cells by canonical cell marker genes grouped all cells into 11 cell populations, including GMPs, common lymphoid precursors, hematopoietic stem and progenitor cells, plasma-blasts, monocytes, neutrophils, osteoclasts, mast cells, plasmacytoid dendritic cells and lymphoid cells (T and B cells) (Fig. 3A,B; Fig. S1A).

Datasets from each genotype group had all of these cell compartments; however, the overall proportion of myeloid cells was higher in *Pstpip2^−/−^* mice than that in WT or *Pstpip2^−/−^Morrbid^−/−^* mice (Fig. 3C). In particular, the *Pstpip2^−/−^* mice had the greatest proportion of osteoclasts (green dots or bar in Fig. 3A-C). To investigate the effects of *Pstpip2* mutation and *Morrbid* deletion on the development states of different cell types, we visualized the cellular stemness of individual cells by the computational tool CytoTRACE. As shown in Fig. 3D, the stemness scores of osteoclasts, GMPs and neutrophils in *Pstpip2^−/−^* mice were enhanced. However, loss of *Morrbid* returned the scores of these myeloid cells to a normal level. We speculate that loss of *Morrbid* changes the landscape of apoptosis of inflammatory myeloid cells, thus changing the stemness of certain immature myeloid cells in the DKO mice. We quantified the stemness and apoptosis scores and observed obvious changes in osteoclasts in *Pstpip2^−/−^* mice compared to those in WT controls (Fig. S2A,B). We also performed bromodeoxyuridine (BrdU) chasing experiments and observed that, compared with WT controls, the *Pstpip2*^−/−^ mutant neutrophils and monocytes manifested an extended half-life (Fig. S3A-D).

Consistent with the results in Fig. 1J,L, scRNA-seq datasets of *Pstpip2^−/−^* mice, along with those of WT and DKO mice, suggested increased inflammatory and ROS metabolic scores (total cells and myeloid-lineage cells were analyzed, respectively; Fig. 3E,F). Moreover, cells from *Pstpip2^−/−^* mice had increased scores for differentiation, proliferation, ubiquitination, endoplasmic reticulum (ER) stress and apoptosis (Fig. 3G). We performed analyses of DEGs in important clusters with different comparisons. The results showed that matrix metalloproteinase genes (*Mmp3* and *Mmp13*) and a serum amyloid gene (*Saa3*) were upregulated in multiple cell populations from *Pstpip2^−/−^* mice compared to those from WT mice (Fig. S1B). In previous studies, high expression of these genes positively correlated with inflammatory disease progression (de Almeida et al., 2022; Ji et al., 2019; Hu and Ecker, 2021; Vallon et al., 2001; Lee et al., 2020; Yao et al., 2023). In contrast, the expression of these genes was significantly reduced in DKO mice (Fig. S1C). These results suggest that the scRNA-seq analysis captured changes in multi-layers of biological processes in *Pstpip2^−/−^* mice compared to WT controls and that such changes were minimized in the DKO mice. Of note, lncRNAs *Malat1* and *Prg4* (encoding proteoglycan 4) were upregulated in DKO mice (Fig. S1C). Previous studies have demonstrated the role of the lncRNA *Malat1* in bone and cartilage diseases and osteogenic differentiation (Zhao et al., 2024; Huang et al., 2023; Alnajjar et al., 2021). Proteoglycan encoded by *Prg4* was reported to function as a cartilage surface lubricant and be involved in repairing articular cartilage damage in mouse models of inducible full-thickness injury (Yao et al., 2023; Massengale et al., 2023).

### Transcriptomic alterations in GMPs, osteoclasts/macrophages and neutrophils

**Fig. 4.**
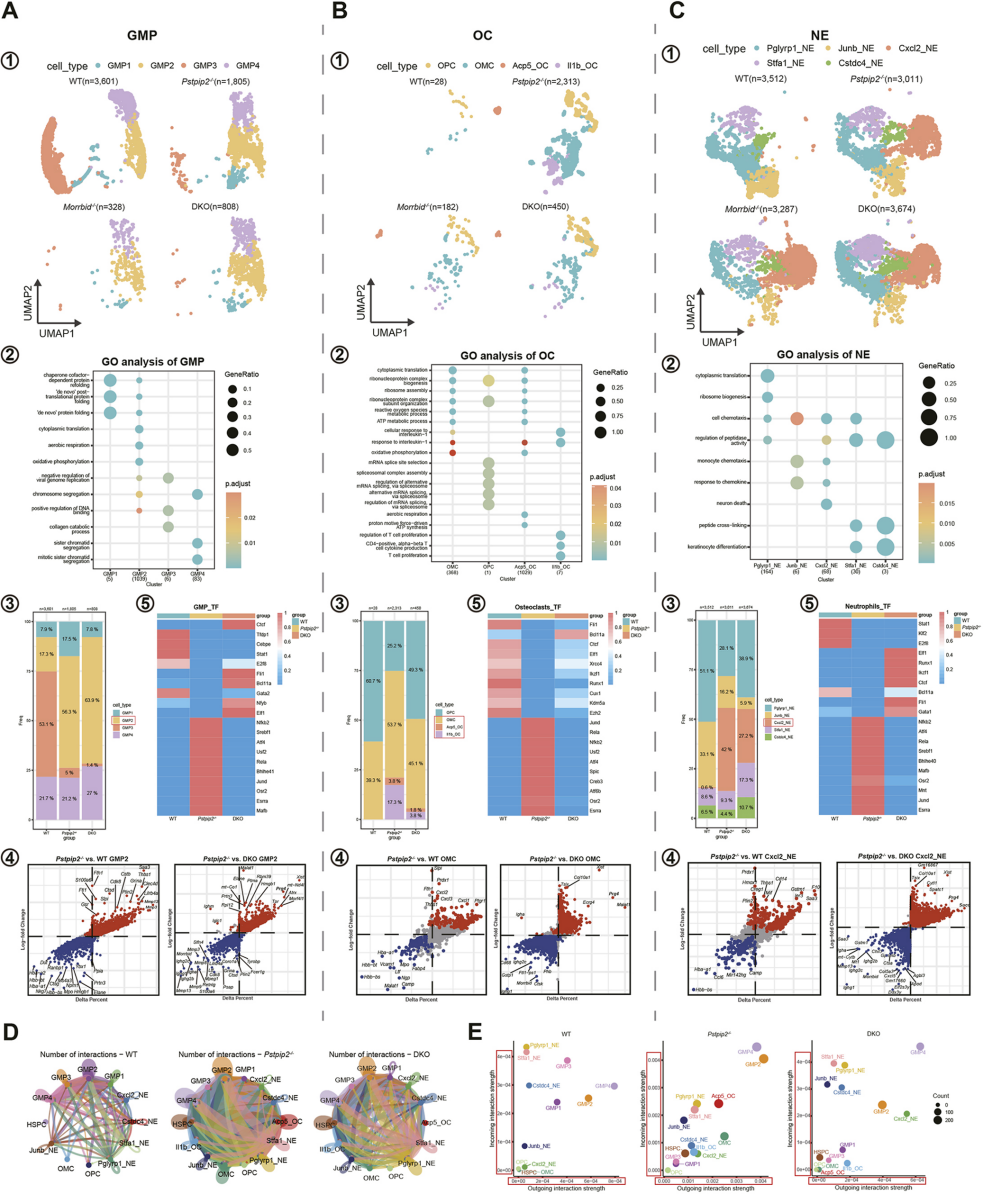
**Characteristics of subsets in GMPs, osteoclasts and neutrophils.** (A-C) Myeloid-linage cells including granulocyte-macrophage-progenitor (GMP), osteoclasts (OC) and neutrophils (NE) were extracted for further analysis. Four genotypes of mice were included: WT, *Pstpip2^−/−^*, *Morrbid^−/−^* and DKO. ① UMAP plots of GMP, OC and NE, respectively; ② Gene Ontology (GO) enrichment analysis of different cell types as indicated; ③ stacked charts of the various cell subsets; ④ scatter plots showing DEGs in indicated comparisons (each point represents a gene); ⑤ heatmap of transcriptional factor regulon activity score (TF-RAS) in GMP, OC and NE, respectively. (D) Circle plots showing cell–cell communications between cell subtypes in the BM scRNA-seq datasets from WT, *Pstpip2^−/−^* and DKO mice. (E) Overall signal intensities based on outgoing (*x*-axis) and incoming (*y*-axis) strengths. Of note, a much higher basal level of signals was determined in *Pstpip2^−/−^* mice than in WT and DKO mice (∼10-fold higher for the scale in *x*- and *y*-axes).

In previous studies, macrophages (including the specialized macrophages, osteoclasts) and neutrophils have been implicated in CRMO. In our scRNA-seq datasets, we focused on three cell types: osteoclasts, neutrophils and their common progenitors GMPs. These cell compartments were extracted and re-clustered for better resolution of their subsets. We annotated a total of four cell subsets in GMPs, four cell subsets in osteoclasts and five cell subsets in neutrophils (Fig. 4A①, B①, C①). Gene Ontology (GO) enrichment analysis was used to understand the function of each cell type. GMPs seemed to be related to the catabolism of collagen. Osteoclasts were highly associated with the response to IL-1 and ROS metabolism. The chemotaxis-related functions of neutrophils were significantly enriched (Fig. 4A②, B②, C②). These functional suggestions indicate key roles of these three compartments of cells in CRMO.

When comparing the proportions of each cell subtype in WT and *Pstpip2^−/−^* mice, we paid attention to the cell subsets with large differences between the two groups, namely subset 2 of GMPs (GMP2), osteoclast-macrophage cells (OMC) and Cxcl2^+^ neutrophils (Cxcl2_NE) (Fig. 4A③, B③, C③, respectively), and carried out further analysis. The DEG analysis showed that inflammation and peroxidation-related genes – such as *Saa3*, *Grina*, *Mmp13* and *Prdx1* – were significantly upregulated, whereas genes involved in bone protection and remodeling, such as *Suco* and *Prg4*, were significantly downregulated, in *Pstpip2^−/−^* compared to WT and DKO mice (Fig. 4A④, B④, C④). Kyoto Encyclopedia of Genes and Genomes (KEGG) enrichment analysis of GMP2 and Cxcl2_NE, and GO analysis of OMC, showed that the pathways and functions of cell metabolism, rheumatoid arthritis and osteoclast differentiation were significantly upregulated in *Pstpip2^−/−^* compared to WT and DKO mice (Fig. S1D,E). The *Pstpip2^−/−^* mice had *Il1b*^+^ osteoclasts (*Il1b*_OC) and *Acp5*^+^ osteoclasts (*Acp5*_OC), which were not present in the WT mice, and the proportion and numbers of these two osteoclast subsets in DKO mice were obviously lower than those in *Pstpip2^−/−^* mice. We therefore concluded that *Il1b*_OC and *Acp5*_OC are very likely the major osteoclast populations that play an osteolytic role in CRMO.

By leveraging the scRNA-seq datasets and by computing the transcriptional factor regulon activity score (TF-RAS) in the pools of GMPs, osteoclasts and neutrophils, we explored the transcription factor activities in the three genetic backgrounds. We found that some transcription factors (including Nfkb2, Rela, Jund, Atf4 and Esrra) had increased TF-RAS in *Pstpip2^−/−^* mice while others (Ctcf, Elf1, Fli1, Bcl11a and Runx1) had greater TF-RAS in the WT and DKO mice (Fig. 4A⑤, B⑤, C⑤).

Further analysis of the pools of *Il1b*_OC and *Acp5*_OC were conducted. As *Acp5*_OC in the DKO could not be enriched owing to the low numbers of cells, we then compared differences in the pool of *Il1b*_OC only. DEG analysis showed that inflammation-related genes, such as *Mmp13*, *Grina* and *S100a8*, were highly expressed in *Pstpip2^−/−^* mice, while an osteoprotective gene, *Prg4*, was significantly upregulated in DKO mice (Fig. S1F). GO enrichment analysis showed that this subset of osteoclasts was associated with immune disorders and chronic inflammatory diseases (Fig. S1F).

We also addressed other important questions about the cell–cell interactions in the pathogenic BM microenvironment in CRMO. By using the computational tool CellChat, we revealed that the number of interactions was obviously increased in the *Pstpip2^−/−^* mice compared to that in the other two groups (Fig. 4D; Fig. S1G). These results suggest disturbed cellular activity in CRMO-like diseased tissues. In the pairwise comparison of the overall signaling patterns (*Pstpip2^−/−^* versus WT or *Pstpip2^−/−^* versus DKO), we also revealed that MIF, VISFATIN (also known as NAMPT), SPP1, CSF3, TGFβ and GDF signaling pathways are activated in *Acp5*_OC of *Pstpip2^−/−^* mice (Fig. S1H). Comparing the differences in signaling pathways in the GMPs from the three groups, we found intense signal for TGFβ, SEMA3, KIT, APRIL (also known as TNFSF13) and BAFF signaling pathways in DKO mice (Fig S1I). In addition, the intensities of the incoming and outgoing interactions (information flow) in *Pstpip2^−/−^* mice were greater than those in WT or DKO mice. GMP2, GMP4 and *Acp5*_OC were the top three cellular compartments with active cell signaling activities (Fig. 4E).

### Experimental validations in *Pstpip2^−/−^* and DKO mice

**Fig. 5.**
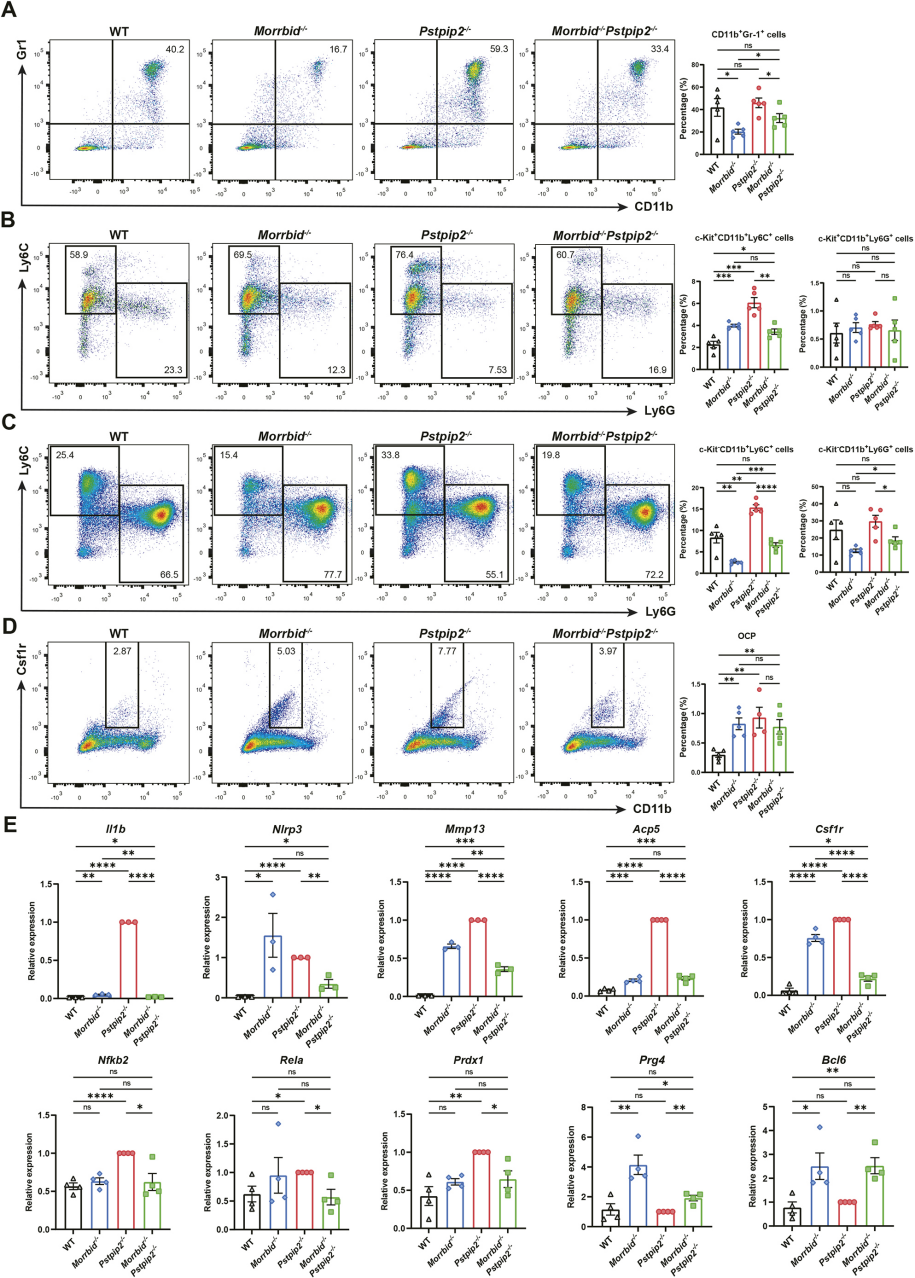
**Experimental analysis using flow cytometry and real-time quantitative PCR (RT-qPCR) for validating findings from scRNA-seq analysis**. (A-D) Representative flow cytometry profiles in BM samples from the four groups of mice (left) and quantification of the results (right): general myeloid cells (Gr-1^+^CD11b^+^) (A); immature monocytes (c-Kit^+^CD11b^+^Ly6C^+^) and immature neutrophils (c-Kit^+^CD11b^+^Ly6G^+^) (B); mature monocytes (c-Kit^−^CD11b^+^Ly6C^+^) and mature neutrophils (c-Kit^−^CD11b^+^Ly6G^+^) (C); osteoclast precursors (OCP; c-Kit^+^CD11b^low^Csf1r^+^) (D). *n*≥4 animals for each group. (E) Expression levels of the indicated genes in hind paw BM cell lysates, determined by RT-qPCR. *n*≥3 animals for each group. Unpaired two-tailed Student's *t*-test, mean±s.e.m.; ns, not significant; *P≤0.05, **P≤0.01, ***P≤0.001, ****P≤0.0001.

The scRNA-seq analysis shown above provides an unbiased and comprehensive understanding of the pathology of CRMO-like disease and the involvement of *Morrbid* in the disease (Figs 3 and 4). To verify the key findings of the scRNA-seq analysis, we performed experiments including immunological blotting, flow cytometry and real-time quantitative PCR (RT-qPCR) assays of BM cells including osteoclast precursors (OCPs). In the BM cell-derived macrophage cellular models, we observed enhanced expression of Nlrp3 (inflammation marker) and lncRNA *Morrbid* (myeloid-lineage pro-survival marker) (Fig. S4A,B). Visualization and quantification of the expression of *Bim* (*Bcl2l11*) and *Morrbid* highlights the robust changes in the *Pstpip2^−/−^* and DKO mice (Fig. S5A-D). The frequencies of general myeloid cells [Gr-1^+^ (also known as GSR^+^)CD11b^+^ (also known as ITGAM^+^)] and OCPs [c-Kit^+^ (also known as KIT^+^)CD11b^low^Csf1r^+^] were all significantly increased, or with a trend for increase, in the BM of *Pstpip2^−/−^* mice compared to that of WT and *Pstpip2^−/−^Morrbid^−/−^* mice (Fig. 5A,D). A similar trend was observed in immature and mature neutrophils (c-Kit^+^/CD11b^+^Ly6G^+^, c-Kit^−^/CD11b^+^Ly6G^+^) and in immature and mature monocytes (c-Kit^+^/CD11b^+^Ly6C^+^, c-Kit^−^/CD11b^+^Ly6C^+^) (Fig. 5B,C).

The expression level of *Il1b* in the hind paw was significantly increased in *Pstpip2^−/−^* mice, consistent with our above ELISA data for IL-1β (Fig. 5E). Reportedly, the Nlrp3 inflammasome is involved in the pathogenesis of CRMO-like disease in *Pstpip2^cmo/cmo^* mice, not only by converting pro-IL-1β to active IL-1β but also by ROS production and cellular stress (Kralova et al., 2020). In our scRNA-seq analysis of cellular transcription factors, we also found that the transcription factor Nfkb2, which is related to the Nlrp3 pathway, was significantly activated in the tissues of hind paws of *Pstpip2^−/−^* mice (Fig. 5E). The expression level of *Nlrp3* in our four groups of mice was measured, which confirmed its important role in CRMO (Fig. 5E). We also examined the expression levels of other inflammation-related genes (e.g. *Mmp13*, *Acp5*, *Csf1r*, *Prdx1*, *Prg4*) and genes encoding representative transcription factors (e.g. *Nfkb2*, *Rela*, *Bcl6*) identified by our scRNA-seq analysis (Fig. 5E). These results were consistent with the findings obtained with single-cell transcriptome analysis of the four groups of mice.

## DISCUSSION

**Fig. 6.**
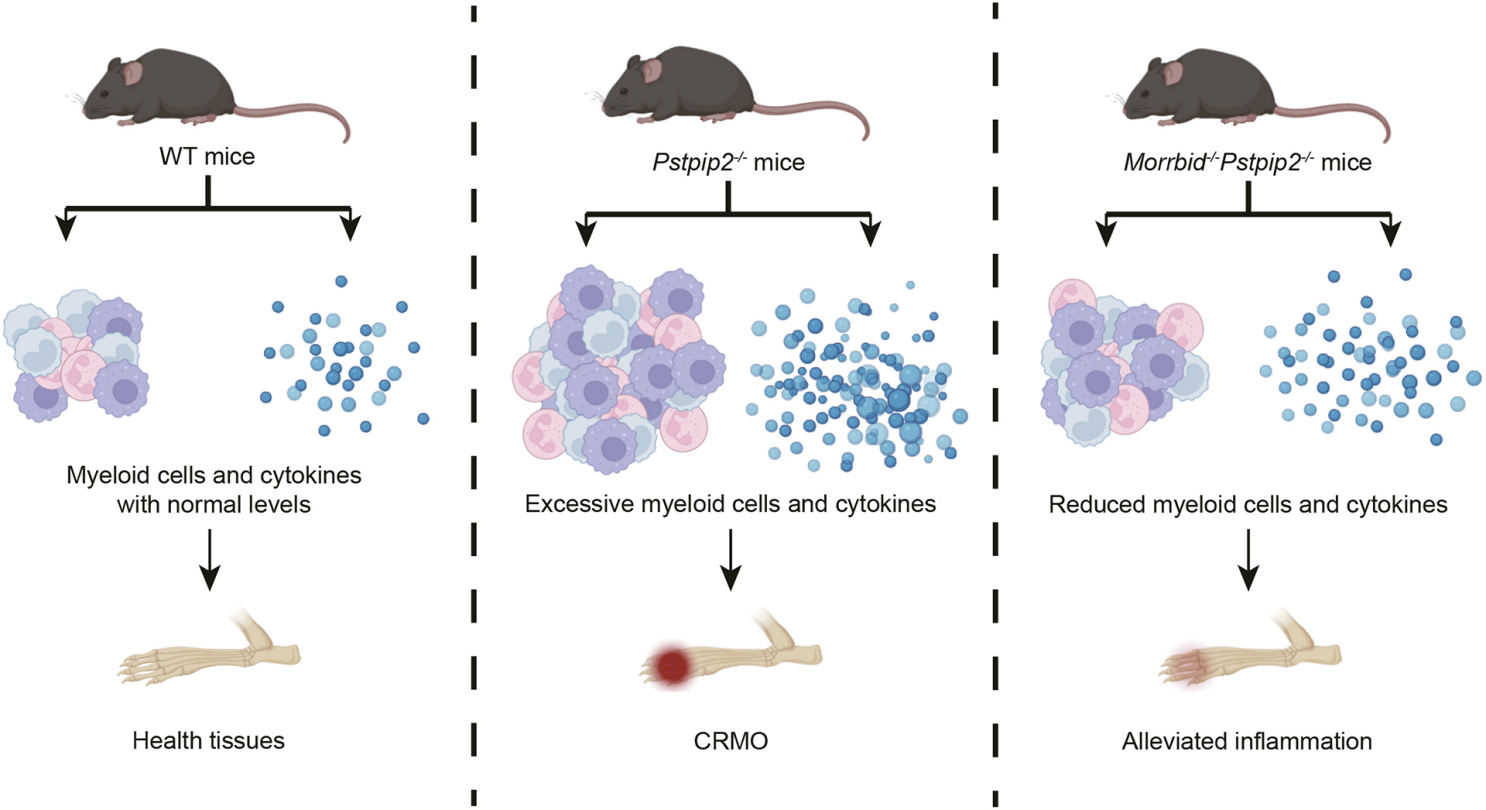
**Summary of the study.** Mouse models with genetic mutations in *Pstpip2* are one of the most important models for studying autoinflammatory diseases (AIDs) including chronic recurrent multifocal osteomyelitis (CRMO). We generated a strain with 5-bp deletion in *Pstpip2* for testing *Morrbid* involvement in AIDs. Our genetic analysis and pathological analysis, including scRNA-seq profiling and flow cytometry validations, demonstrate that loss of *Morrbid* mitigates osteomyelitis and that *Morrbid* is a potential target for anti-inflammation.

Although *PSTPIP2* has not been directly implicated in human AIDs, the murine mutant strain *Pstpip2^cmo/cmo^* is widely used for testing inflammation regulation and as a classic model of CRMO. In the present study, we describe a new *Pstpip2* knockout strain and confirmed that *Pstpip2* loss of function causes a CRMO-like disease in mice. More importantly, we demonstrated that loss of *Morrbid* inhibited the occurrence of the disease, and this inhibition is likely through multiple layers of mechanisms (i.e. the numbers of inflammatory myeloid cells and production of IL-1β). Our results not only demonstrated the pathogenic link between hyperactivation of lncRNA *Morrbid* and occurrence of AIDs, but also provided a single-cell atlas to reveal CRMO pathogenesis (see the graphical summary in Fig. 6).

The previously reported *Pstpip2* mutants are germline based and display very similar appearance of swollen toes at the hind paws. Interestingly, according to previous reports, the onsets of the swollen toes are slightly different and can be classified into three groups: Group-1, as early as 4 weeks [see Liao et al. (2015)]; Group-2, 6-7 weeks [see Pavliuchenko et al. (2024)]; Group-3, as late as 8 weeks [see Pavliuchenko et al. (2022)].

The differences between Group-1 and Group-2 may be caused by genetic background [as indicated by Pavliuchenko et al. (2024)]. However, the differences between Group-2 and Group-3 may be caused by residual mutant protein isoforms (Pstpip2*) in Group-3 [as indicated by Pavliuchenko et al. (2022)].

In addition to the highly similar appearance of paw swelling, the histological pathology and classical molecular pathology are almost identical or comparable between the groups. They could be measured or observed as follows: (1) ROS production (Fig. 1L; Kralova et al., 2020); (2) lymph node enlargement (Fig. 1E; Gurung et al., 2016); (3) inflamed hind paws (Fig. 1C,D; Chitu et al., 2012); (4) profound neutrophil infiltration (Fig. 1H; Pavliuchenko et al., 2022); (5) profound osteolytic lesions (Fig. 1K; Pavliuchenko et al., 2022); (6) elevation of chemokine MIP-1α (CCL3) (Fig. 1J; Pavliuchenko et al., 2022); (7) upregulation ofupregulation of proinflammatory cytokine IL-1β (Fig. 1J; Pavliuchenko et al., 2022; Gurung et al., 2016); (8) increased CD11b/Ly6C^+^ (monocyte marker) cells (Fig. 5B,C; Kralova et al., 2020).

Chronic non-bacterial osteomyelitis is an inflammation of bone tissue without bacterial infection. When it is active and occurs in multiple foci, it is referred to as CRMO (Hofmann et al., 2017). Patients with CRMO usually present with recurrent fever and bone pain, and swelling and deformity may occur in the affected area (Cox and Ferguson, 2018). Mice with *Pstpip2* mutation develop CRMO lesions and show a massive increase in myeloid cells at the lesion location. The overproduction of osteoclasts causes bone damage at the lesion site and severely destroys the basic structure of bone. Massive infiltration of neutrophils and monocytes/macrophages leads to excess release of proinflammatory cytokines (Chitu et al., 2012). These alterations in cell compartments were readily revealed by our scRNA-seq analysis. Biased myelogenesis is also indicated by the perpetual cycle for the progression of CRMO in the *Pstpip2^cmo/cmo^* mice. However, the efficacy of depleting inflammatory cells in CRMO had not been tested using DKO mutants *Pstpip2^−/−^Morrbid^−/−^* until this study.

*Morrbid* is a lncRNA and controls myeloid cell lifespan. Myeloid cells with loss of *Morrbid* exhibit increased apoptosis (Kotzin et al., 2016). Using *Pstpip2^−/−^Morrbid^−/−^* mice, we confirmed that deletion of *Morrbid* in *Pstpip2^−/−^* mice significantly reduced the number of myeloid cells at the lesion site, lowered inflammatory cytokines and ROS levels, and alleviated bone damage (Fig. 6). The high conservation of *Morrbid* between humans and mice suggests the possibility of targeting *Morrbid* to inhibit the development of CRMO. How to effectively and specifically target *Morrbid* will be a challenge to overcome in future studies.

Interestingly, as shown in Fig. 3E,F and Fig. 5E, compared to that in WT controls, the phenotype observed in *Morrbid*^*−/−*^ mice is unexpected and contradictory to our canonical understanding of the regulatory role of *Morrbid* in myelogenesis according to what has been published in the literature (Kotzin et al., 2016; Cai et al., 2018). Especially in mature cells, the function of *Morrbid* might not just be limited to regulating the lifespan of neutrophils, monocytes and eosinophils. Additional experimental and dynamic assays are required to test whether and how the lncRNA *Morrbid* also intrinsically regulates the differentiation and maturation of phagocytic myeloid cells and their cellular homeostasis.

Although pathogenic variants of the *PSTPIP2* gene have not yet been found in patients with CRMO, the mouse *Pstpip2* mutants serve as an important disease model for studying autoinflammation. The phenotype produced by the loss of *Pstpip2* in mice is very similar to the symptoms of patients with CRMO. As shown in Figs 2-5, we have demonstrated that *Morrbid* can serve as a new target for inhibiting or slowing down the progression of CRMO and even other AIDs caused by the abnormal expansion of myeloid cells. We also conjecture that *Morrbid* could be a target for potential anti-sense oligonucleotide (ASO) drugs, and the progression of CRMO can be blocked or alleviated by anti-*Morrbid* ASO drugs. In a recent work on chronic granuloma formation, we also reported that loss of *Morrbid* mitigated such inflammatory structure formation (Yu et al., 2025). In a preprint, we also observed that loss of *Morrbid* mitigated a newly identified AID, VEXAS (Dong et al., 2025).

In addition, the main cell type driving the initiation of CRMO in *Pstpip2^−/−^* (or *Pstpip2^cmo/cmo^*) mice remains controversial. The prominent role of myeloid cells in the progression of CRMO has been observed here and in previous studies. Authors of the previous studies held the view that neutrophil or monocyte lineage drives the initiation of CRMO (Chitu et al., 2012; Kralova et al., 2020; Lukens et al., 2014; Chitu et al., 2009). Our study, with insights from scRNA-seq analysis, confirmed an increased frequency of multiple compartments of myeloid cells, especially neutrophils and osteoclasts, in the lesions and peripheral blood of mice with CRMO.

Through scRNA-seq analysis of the four genotypes of mice, *Pstpip2^−/−^* mice showed increased inflammatory levels and ROS production, significantly upregulating the expression of genes that promote inflammation, consistent with previous studies suggesting that loss of NOX2 activity mitigates CRMO symptoms (Chitu et al., 2012; Cassel et al., 2014; Yao et al., 2022; Kralova et al., 2020). Furthermore, *Pstpip2^−/−^* mice exhibited enhanced intercellular communication and increased activity of transcription factors related to the NF-κB pathway. Deficiency of *Morrbid* resulted in a comprehensive inhibitory effect in these aspects. However, further studies using conditional knockout mice are required to determine which type of myeloid cell plays a dominant role in the CRMO initiation and the primary cell type affected by deletion of *Morrbid*.

In conclusion, combining single-cell analysis and genetic murine models, this study shows that CRMO involves multiple layers of inflammatory myeloid cells and provides preclinical evidence supporting that *Morrbid* could be a therapeutic target for AIDs including chronic osteomyelitis.

## MATERIALS AND METHODS

### Animals

We used WT, *Morrbid^−/−^*, *Pstpip2^−/−^*, and *Pstpip2^−/−^Morrbid^−/−^* mice in this study. All mice were on C57BL/6J background. *Morrbid^+/−^* and *Pstpip2^+/−^* mice were generated by and purchased from Cyagen Biosciences (Suzhou, China). *Morrbid^+/−^* mice were generated by knockout of six exons of *Morrbid*. *Pstpip2^+/−^* mice were generated by a 5-bp deletion in *Pstpip2* Exon 2. Homozygous KO mice were generated by self-breeding of *Pstpip2^+/−^* or *Morrbid^+/−^* mice. DKO *Pstpip2^−/−^Morrbid^−/−^* mice were generated by crossing *Morrbid^+/−^* mice with *Pstpip2^+/−^* mice. All animal experiments were performed in accordance with protocols approved by the Animal Care and Use Committee of Tianjin Medical University.

### Histopathology

The hind paws were fixed in 4% paraformaldehyde (p1110, Solarbio) for 24 h and decalcified in EDTA Decalcified Solution (BL616B, Biosharp) for 2-3 weeks, followed by paraffin embedding and histological cutting. The slice thickness was 4 μm. H&E staining was used to detect the infiltration of immune cells and inflammatory granulomas. For IHC staining, sections were stained with anti-Ly6G (E6Z1T) rabbit monoclonal antibody (87048, Cell Signaling Technology) and counterstained with Hematoxylin. H&E staining and IHC staining were performed using a BOND automatic system (Leica), and slides were scanned in Aperio (Leica). Image postprocessing and analysis were done in Aperio ImageScope software (Leica).

For TRAP staining, paraffin embedding and tissue sectioning were performed as previously described (Cassel et al., 2014). Slides were stained with a TRAP staining kit (G1050, Servicebio) according to the operating standard, and scanned in a 3DHISTECH scanner (DANJIER, China), and images were processed using 3DHISTECH SlideViewer software (DANJIER).

### Hemogram detection of peripheral blood

Heparinized capillary tubes (2501, KIMBLE) were used to access the fundi of mouse eyes and rotated to cut the venous plexus. After sufficient blood had been drawn, it was transferred to centrifuge tubes with anticoagulant (110403015, BKMAM). The number and frequency of blood cells were detected by an automatic blood cell analyzer (URIT, China).

### Micro-CT

Hind paws were scanned by *in vivo* micro-CT (Skyscan 1276, Bruker). Scanning parameters were as follows: voltage, 50 kV; current, 200 μA; filter, 0.25 mm aluminum; pixel size, 9 mm; exposure time, 650-800 ms; rotation step, 0.4° for 180° total; object to source distance, 81.719 mm; camera to source distance, 158.176 mm; time of scanning, 7 min. Reconstruction of virtual slices was performed in NRecon software 1.7.3 (Bruker) with setup for smoothing=3, ring artifact correction=6 and beam hardening correction=30%. Intensities of interest for reconstruction were in the range of 0.0030-0.1500. For reorientation of virtual slices to the same orientation, DataViewer 1.5.4 software (Bruker) was used. CTvox 3.3.0 (Bruker) was used for micro-CT image processing. Quantification analysis was performed using CT-Analyser (CTAn) 1.17.7 (Bruker).

### ROS detection

To assess ROS production *in vivo*, mice were intraperitoneally (i.p.) injected with luminescence reporter L-012 (120-04891, FUJIFILM Wako) at a final concentration of 25 mg/kg dissolved in PBS with a concentration of 20 mmol/l. Luminescence signal was acquired by IVIS Spectrum (PerkinElmer), with the exposure parameters set to automatic. The quantification of photon counts was performed in Living Image Software (PerkinElmer).

### ELISA

Hind paw tissue was cut into small pieces and homogenized with a tissue homogenizer (60 Hz, 60 s) in 1 ml RIPA lysis buffer (E122-01, Genstar) containing 1 mM PMSF (B111-01, Genstar). After two rounds of centrifugation (each 16,101 ***g***, 10 min, 4°C), the lysates were snap frozen in liquid nitrogen and stored at −80°C.

The frozen tissue homogenates described above were thawed, and total protein concentration was measured by a Nanodrop spectrophotometer (Thermo Fisher Scientific). ELISA was performed according to the manufacturer's instructions using a Mouse IL-1β ELISA Kit (EK201B, MULTI SCIENCES) and Mouse CCL3/MIP-1a ELISA Kit (EK261, MULTI SCIENCES) to detect the concentrations of IL-1β and CCL3/MIP-1α, respectively, in the tissue homogenates.

### Flow cytometry

The BM cells were flushed out with FACS buffer (2% fetal bovine serum in PBS). Single-cell suspensions were treated with red blood cell lysis buffer (C3702, Beyotime), stained and analyzed using LSRFortessa (BD Biosciences). For myeloid cell analysis, staining was performed with antibodies against CD11b (101224, BioLegend) and Gr-1 (108408, BioLegend). For monocyte and neutrophil analysis, cells were incubated with biotinylated antibodies against c-Kit (105812, BioLegend), CD11b, Ly6C (128024, BioLegend) and Ly6G (127608, BioLegend). For OCP analysis, staining was performed with antibodies against c-Kit, CD11b and Csf1r (135511, BioLegend).

### RT-qPCR

Hind paws were cut into small pieces and homogenized in 1 ml TRIzol using a tissue homogenizer (60 Hz, 60 s). Total RNA was isolated from the hind paws with TRIGene (P118-05, Genstar) according to the manufacturer's instructions. In brief, 200 μl chloroform was added to 1 ml TRIGene, and the samples were incubated at room temperate for 5 min following vertexing. After centrifugation, the aqueous phase was transferred to a new DNase/RNase-free tube (BIOFIL), and equal volumes of isopropanol were added. After incubation at room temperature for 10 min, the RNA was pelleted by centrifugation, and then the RNA was washed two times in 75% ethanol before resuspension in DEPC water. 1 μg of RNA was reverse transcribed to cDNA using StarScript III RT MasterMix (A233, Genstar) according to the manufacturer's instructions. RT-qPCR was performed using 2×RealStar Power SYBR qPCR Mix (A314, Genstar), and samples were run on a LightCycler^®^ 480 Instrument II (Roche). For each sample, transcript levels of tested genes were normalized to the expression level of the gene *Actb*. Oligonucleotides for RT-qPCR analsyis are provided in Table S1.

### Bulk RNA-seq analysis

For RNA extraction, total RNA was extracted from the tissue using TRIzol^®^ Reagent (P118-05, GenStar) according to the manufacturer's instructions. RNA quality was determined by a 5300 Bioanalyzer (Agilent) and quantified using the ND-2000 (NanoDrop Technologies). Only high-quality RNA sample [optical density at 260/280 nm (OD260/280)=1.8-2.2, OD260/230≥2.0, RNA integrity number≥6.5, 28S:18S≥1.0] was used to construct the sequencing library.

For library preparation and sequencing, RNA purification, reverse transcription, library construction and sequencing were performed at Shanghai Majorbio Bio-pharm Biotechnology Co., Ltd. (Shanghai, China) according to the manufacturer's instructions (Illumina). The RNA-seq transcriptome library was prepared following Illumina^®^ Stranded mRNA Prep using 1 μg total RNA. First, mRNA was isolated according to polyA selection method by oligo(dT) beads (R0075, Beyotime) and then fragmented by fragmentation buffer. Second, double-stranded cDNA was synthesized using a SuperScript double-stranded cDNA synthesis kit (Invitrogen) with random hexamer primers (Illumina). Then the synthesized cDNA was subjected to end repair, phosphorylation and ‘A’ base addition according to Illumina's library construction protocol. Libraries were size selected for cDNA target fragments of 300 bp on 2% low-range ultra agarose followed by PCR amplification using Phusion DNA polymerase (NEB) for 15 PCR cycles. After quantification by Qubit 4.0 (Thermo Fisher Scientific), the paired-end RNA-seq library was sequenced with a NovaSeq 6000 sequencer (2×150 bp read length).

For quality control and read mapping, the raw paired-end reads were trimmed and quality controlled by fastp with default parameters. The clean reads were separately aligned to reference genome with orientation mode using HISAT2 software (v2.2.1). The mapped reads of each sample were assembled by StringTie (v2.2.1) in a reference-based approach.

For differential expression analysis and functional enrichment, to identify DEGs between two different samples, the expression level of each transcript was calculated according to the transcripts per million reads (TPM) method. RSEM (v1.3.3) was used to quantify gene abundances. Essentially, differential expression analysis was performed using DESeq2 (v1.38.3) or DEGseq (v1.44.0). DEGs with |log2 fold change|≥1 and false discovery rate (FDR)≤0.05 (DESeq2) or FDR≤0.001 (DEGseq) were considered to be significantly DEGs. In addition, functional enrichment analysis, including GO and KEGG, was performed to identify which DEGs were significantly enriched in GO terms and metabolic pathways at Bonferroni-corrected *P*≤0.05 compared with the whole-transcriptome background. GO functional enrichment and KEGG pathway analysis were carried out by Goatools (v1.3.11) and KOBAS (v3.0), respectively.

### scRNA-seq and analysis of the scRNA-seq datasets

#### Cell preparation

After harvesting, tissues were washed in ice-cold RPMI1640 and dissociated using Collagenase II (9001-12-1, V900892-100MG, Sigma-Aldrich) and DNase I (9003-98-9, DN25-1G, Sigma-Aldrich). Cell count and viability were estimated using a fluorescence cell analyzer (Countstar^®^ Rigel S2) with Acridine Orange/propidium iodide reagent after removal of erythrocytes (R1010, Solarbio). Finally, fresh cells were washed twice in the RPMI1640 and then resuspended at 1×10^6^ cells per ml in 1×PBS and 0.04% bovine serum albumin.

#### scRNA-seq library construction and sequencing

scRNA-seq libraries were prepared using a SeekOne^®^ Digital Droplet Single Cell 3′ library preparation kit (K00202, SeekGene). Briefly, appropriate numbers of cells were mixed with reverse transcription reagent and then added to the sample well in a SeekOne^®^ chip. Subsequently, barcoded hydrogel beads (K00202-0802, SeekGene) and partitioning oil were dispensed into corresponding wells separately in the chip. After emulsion droplet generation, reverse transcription was performed at 42°C for 90 min and inactivated at 80°C for 15 min. Next, cDNA was purified from broken droplets and amplified in a PCR reaction. The amplified cDNA product was then cleaned, fragmented, end repaired, A-tailed and ligated to a sequencing adaptor (K00202-0204, SeekGene). Finally, indexed PCR was performed to amplify the DNA representing the 3′ polyA part of expressing genes, which also contained the cell barcode and unique molecular index. The indexed sequencing libraries were cleaned up with SPRI beads (B23318, Beckman Coulter), and analyzed by a Qubit (Q33226, Thermo Fisher Scientific) and 4200 TapeStation (G2991BA, Agilent). The libraries were then sequenced on an Illumina NovaSeq 6000 with PE150 read length.

#### Identifying the marker genes of single cells

The FindAllMarkers function of Seurat (v4.2.1) was used to identify marker genes of single cells (Slovin et al., 2021). Aggregation-mediated cell clusters were identified based on DEGs exhibiting log fold changes (logFCs) and aggrephagy-related genes.

#### Analysis of transcription factors

SCENIC was employed as a computational tool to simultaneously reconstruct gene regulatory networks and identify stable cell states from scRNA-seq data (Kumar et al., 2021). The inference of the gene regulatory network was based on co-expression analysis and DNA motif analysis, followed by the examination of network activity in individual cells to determine their cellular status (Kumar et al., 2021; Van de Sande et al., 2020). We analyzed transcription factors using the pySCENIC package (Schmitt et al., 2023).

#### Cell–cell communication analysis

The signaling inputs and outputs among the different cell types and aggrephagy-mediated cell clusters were assessed using the CellChat package (Jin et al., 2021). The netVisual_circle function was employed to evaluate the strength of cell‒cell communication networks within specific subsets of cells (Jin et al., 2021).

#### Pseudotime analysis

The core concept of CytoTRACE is that cells with a high level of differentiation express fewer genes, whereas cells with a low level of differentiation express more genes (Gulati et al., 2020). We used the CytoTRACE package to predict the differentiation status of cell subsets.

### Statistical analysis

*P*-values were calculated in GraphPad Prism 9. Comparisons between two groups were performed by unpaired *t*-test (two-tailed). Comparison of multiple groups was performed by two-way ANOVA with Tukey-Kramer or Bonferroni multiple comparisons test. Most of experiments in this study were repeated two or three times independently, and representative data are shown. *n* in figure legends indicates the number of experiment repeats; points in column scatter plots represent biological replicates.

## Supplementary Material

10.1242/dmm.052176_sup1 Supplementary information
