# Supplementary material for: Disruption of Morrbid alleviates autoinflammatory osteomyelitis in Pstpip2-deficient mice
Source: Dis Model Mech. 2025 Jul 7;18(7):dmm052176. doi: 10.1242/dmm.052176 (PMC12309896; doi:10.1242/dmm.052176)
Supplement: Supplementary information [file dmm-18-052176-s1.pdf]

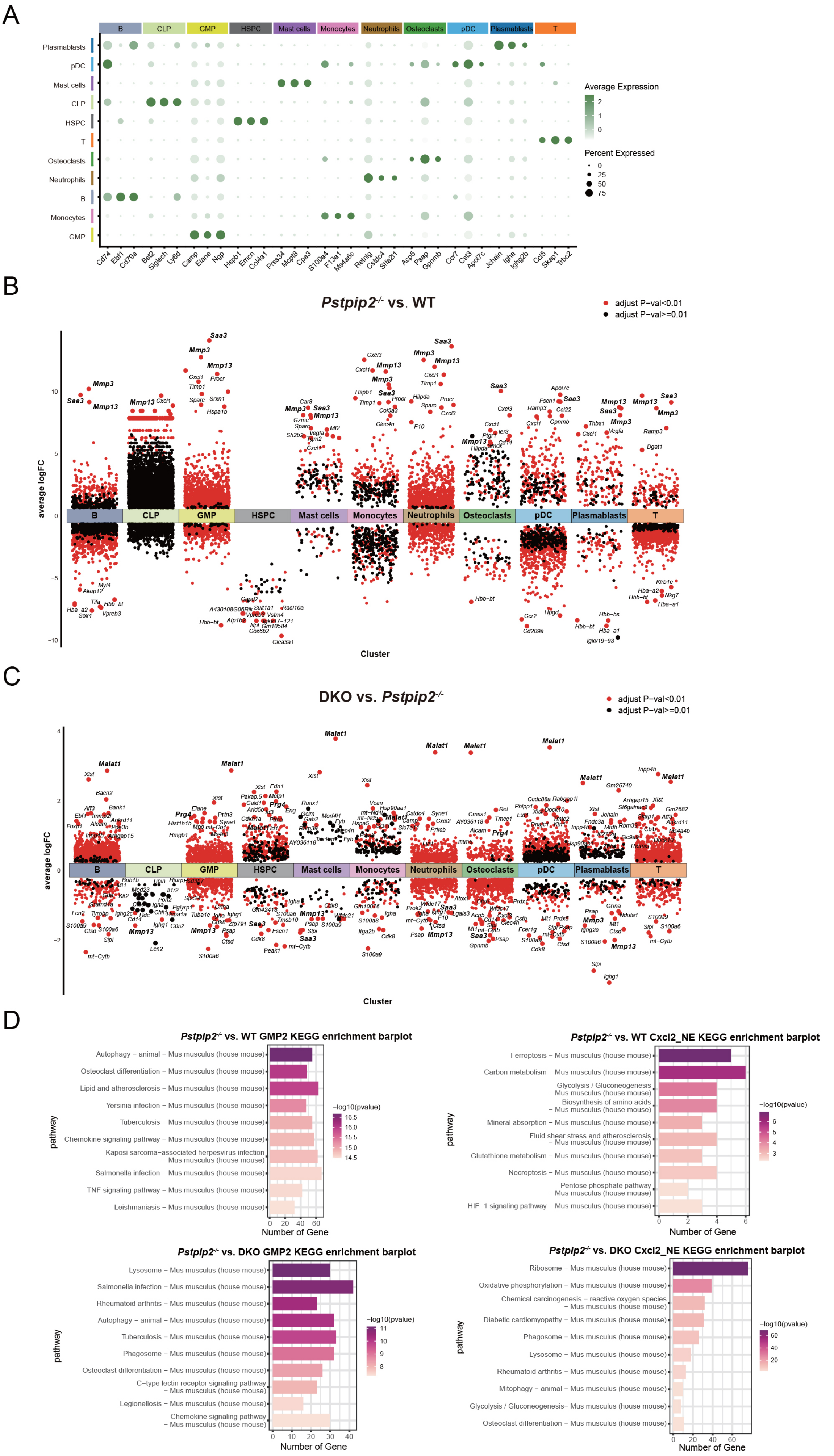
A
B
CLP
GMP
HSPC
Mast cells
Monocytes
Neutrophils
Osteoclasts
pDC
Plasmablasts
T
Average Expression
Percent Expressed
B
Pstpip2-/- vs. WT
adjust P-val<0.01
adjust P-val>=0.01
average logFC
Cluster
C
DKO vs. Pstpip2-/-
D
Pstpip2-/- vs. WT GMP2 KEGG enrichment barplot
Autophagy - animal - Mus musculus (house mouse)
Osteoclast differentiation - Mus musculus (house mouse)
Lipid and atherosclerosis - Mus musculus (house mouse)
Yersinia infection - Mus musculus (house mouse)
Tuberculosis - Mus musculus (house mouse)
Chemokine signaling pathway - Mus musculus (house mouse)
Kaposi sarcoma-associated herpesvirus infection - Mus musculus (house mouse)
Salmonella infection - Mus musculus (house mouse)
TNF signaling pathway - Mus musculus (house mouse)
Leishmaniasis - Mus musculus (house mouse)
pathway
Number of Gene
-log10(pvalue)
Pstpip2-/- vs. WT Cxcl2_NE KEGG enrichment barplot
Ferroptosis - Mus musculus (house mouse)
Carbon metabolism - Mus musculus (house mouse)
Glycolysis / Gluconeogenesis - Mus musculus (house mouse)
Biosynthesis of amino acids - Mus musculus (house mouse)
Mineral absorption - Mus musculus (house mouse)
Fluid shear stress and atherosclerosis - Mus musculus (house mouse)
Glutathione metabolism - Mus musculus (house mouse)
Necroptosis - Mus musculus (house mouse)
Pentose phosphate pathway - Mus musculus (house mouse)
HIF-1 signaling pathway - Mus musculus (house mouse)
Pstpip2-/- vs. DKO GMP2 KEGG enrichment barplot
Lysosome - Mus musculus (house mouse)
Salmonella infection - Mus musculus (house mouse)
Rheumatoid arthritis - Mus musculus (house mouse)
Autophagy - animal - Mus musculus (house mouse)
Tuberculosis - Mus musculus (house mouse)
Phagosome - Mus musculus (house mouse)
Osteoclast differentiation - Mus musculus (house mouse)
C-type lectin receptor signaling pathway - Mus musculus (house mouse)
Legionellosis - Mus musculus (house mouse)
Chemokine signaling pathway - Mus musculus (house mouse)
Pstpip2-/- vs. DKO Cxcl2_NE KEGG enrichment barplot
Ribosome - Mus musculus (house mouse)
Oxidative phosphorylation - Mus musculus (house mouse)
Chemical carcinogenesis - reactive oxygen species - Mus musculus (house mouse)
Diabetic cardiomyopathy - Mus musculus (house mouse)
Phagosome - Mus musculus (house mouse)
Lysosome - Mus musculus (house mouse)
Rheumatoid arthritis - Mus musculus (house mouse)
Mitophagy - animal - Mus musculus (house mouse)
Glycolysis / Gluconeogenesis - Mus musculus (house mouse)
Osteoclast differentiation - Mus musculus (house mouse)

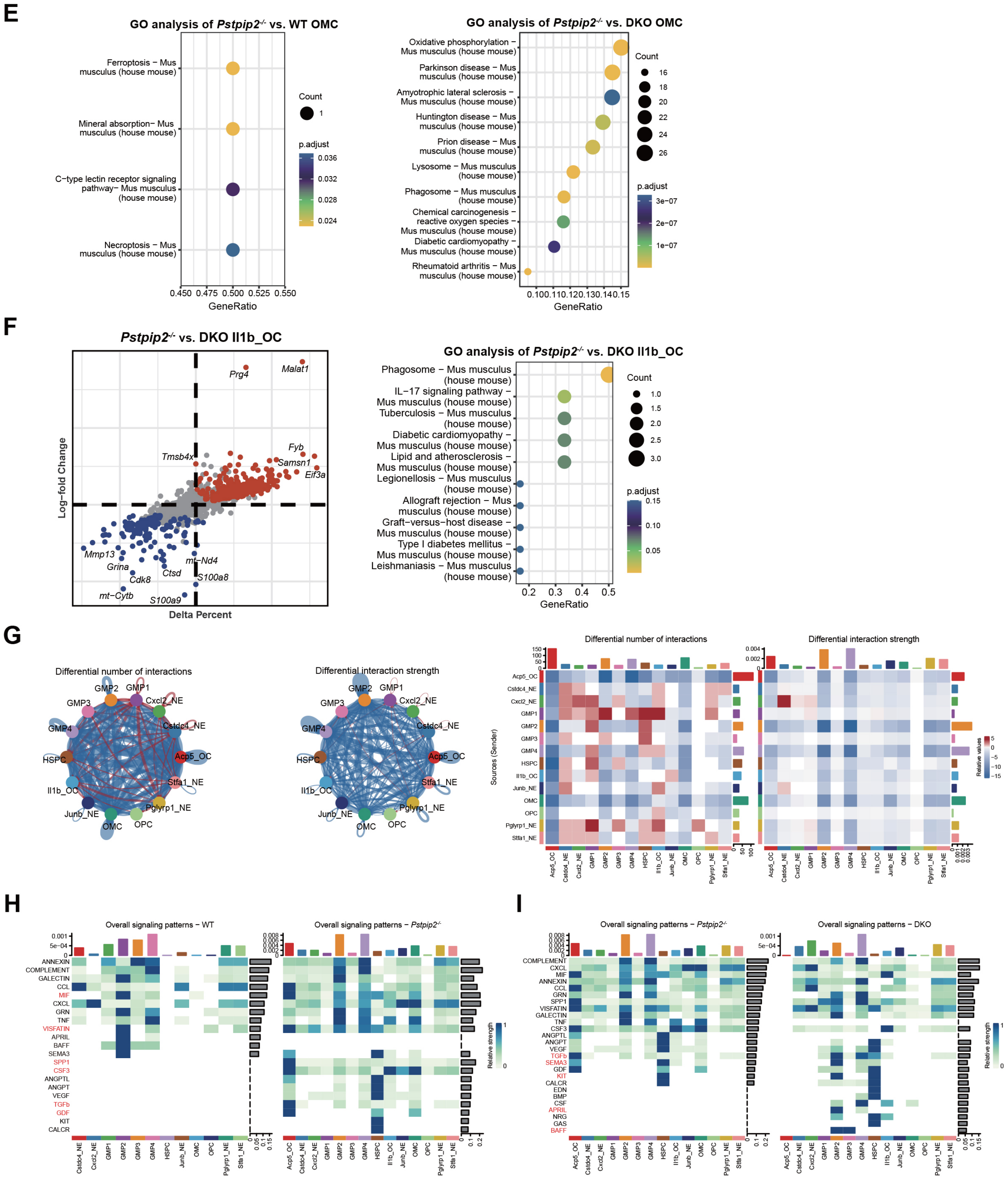

Disease Models & Mechanisms • Supplementary information

J

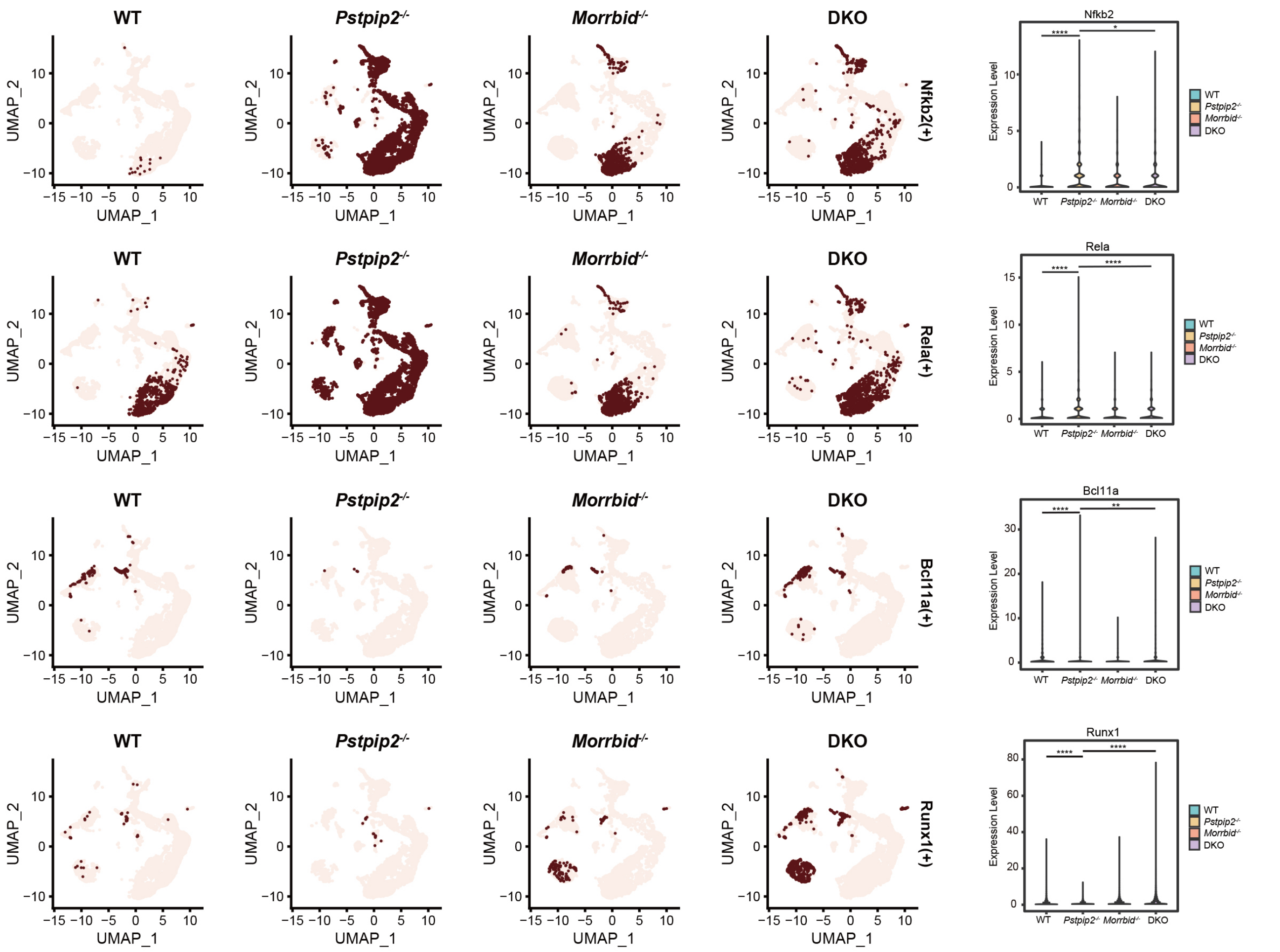

K

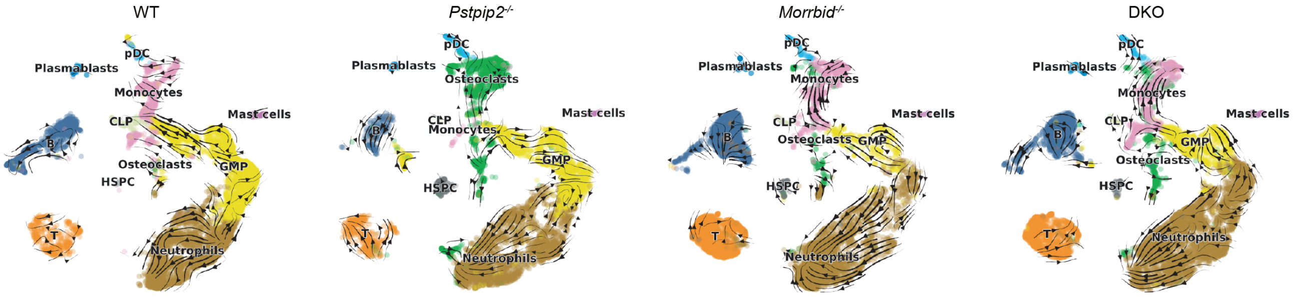

Disease Models & Mechanisms • Supplementary information

L

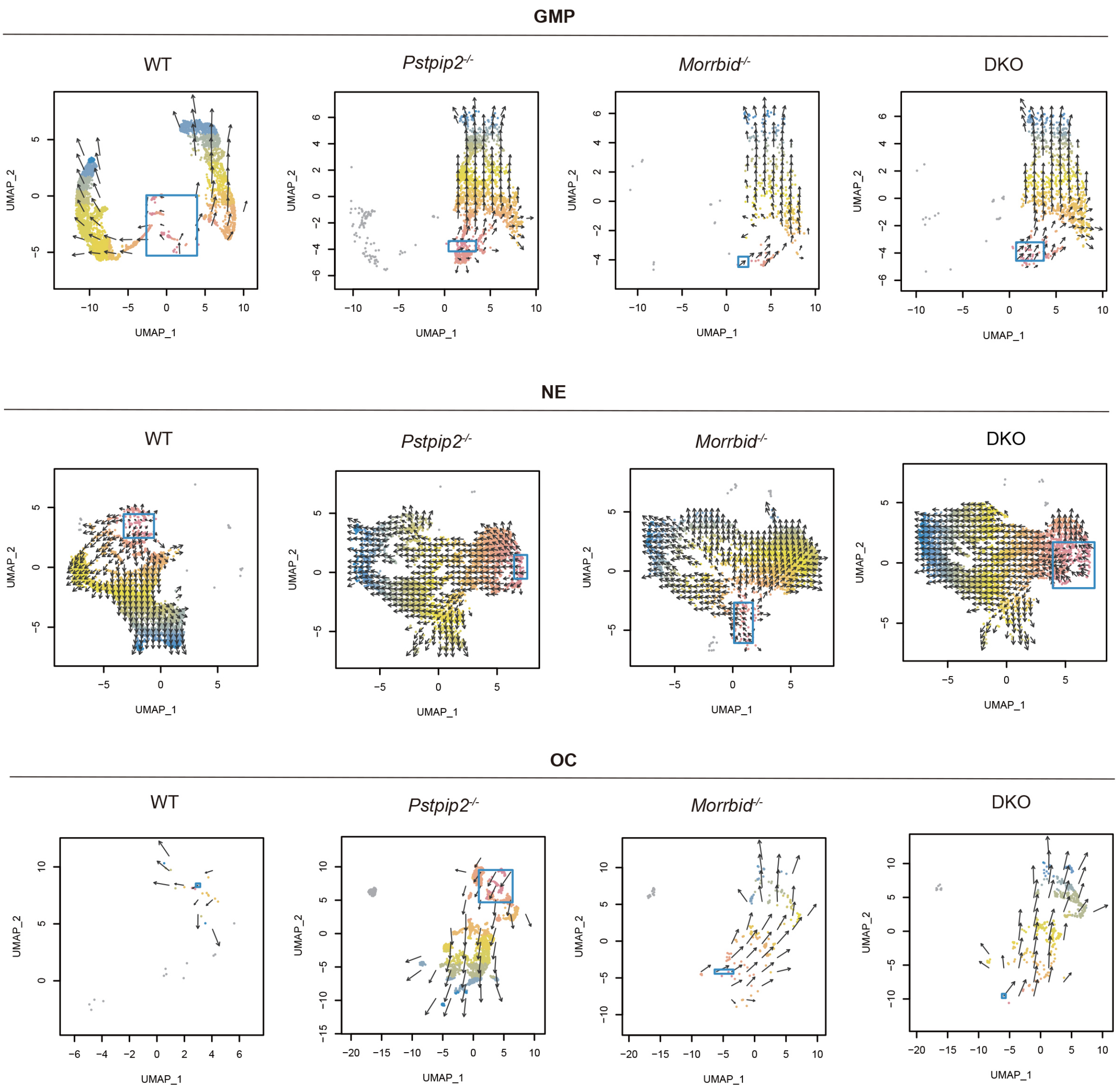

Fig. S1. Additional computational analysis of scRNA-seq datasets from the four genotypes of mice.

(A) Expression profiles of marker genes used for annotating the BM cells in the scRNA-seq datasets.

(B-C) Volcano plots showing DEGs in each cell-type compartment from *Pstpip2*$^{-/-}$ mice compared with that from WT and DKO mice.

(D) KEGG enrichment analysis of GMP2 and Cxcl2_NE among different groups.

(E) GO enrichment analysis of osteoclast/monocytes (OMC) among different groups.

(F) Scatter plots showing DEGs in the indicated comparison.

Disease Models & Mechanisms • Supplementary information

(G) Circle plot (left) and heatmap (right) of the relative number and strength of interactions in DKO compared with *Pstpip2*$^{-/-}$ mice; the up-regulated intercellular interactions are shown in red; the down-regulated intercellular interactions are represented in blue.
(H-I) Heatmap for overall signal patterns of WT compared with *Pstpip2*$^{-/-}$ mice; and for *Pstpip2*$^{-/-}$ mice compared with DKO mice.
(J) Expression (left) and quantification (right) of representative transcription factors in the four genotype mice. Transcriptional factors Nfkb2, Rela, Bcl11a and Runx1 are included. **(K)** RNA velocity in all BM cells from the four groups of mice.
(L) Developmental trajectories of GMP, NE and OC in four groups of mice. Developmental initiation points are marked by blue boxes.

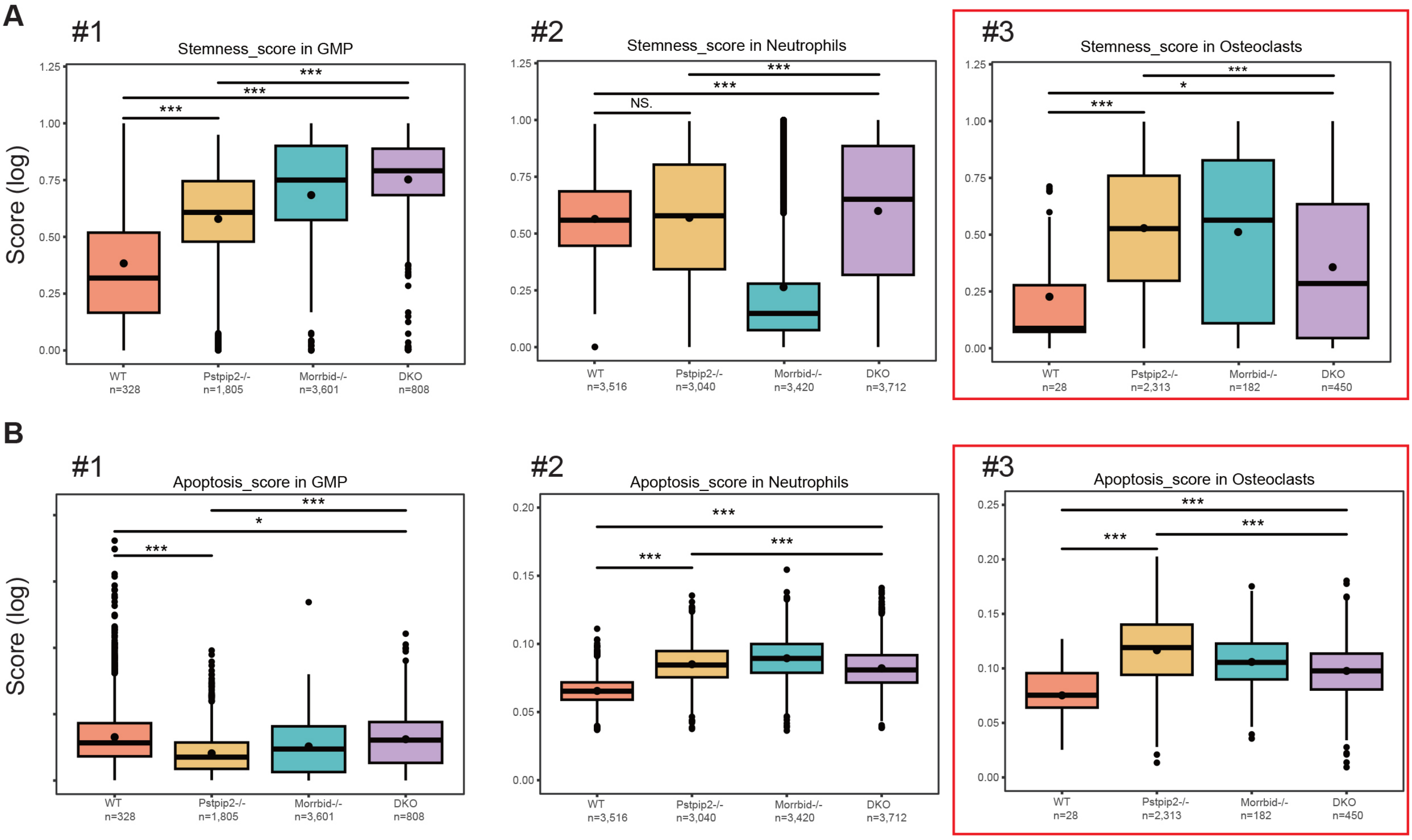

Fig. S2. Scoring the stemness and apoptosis activity in the three pools of myeloid cells: GMP, Neutrophils and Osteoclast.

(A) Scores of cellular stemness.

(B) Scores of Apoptosis.

Disease Models & Mechanisms • Supplementary information

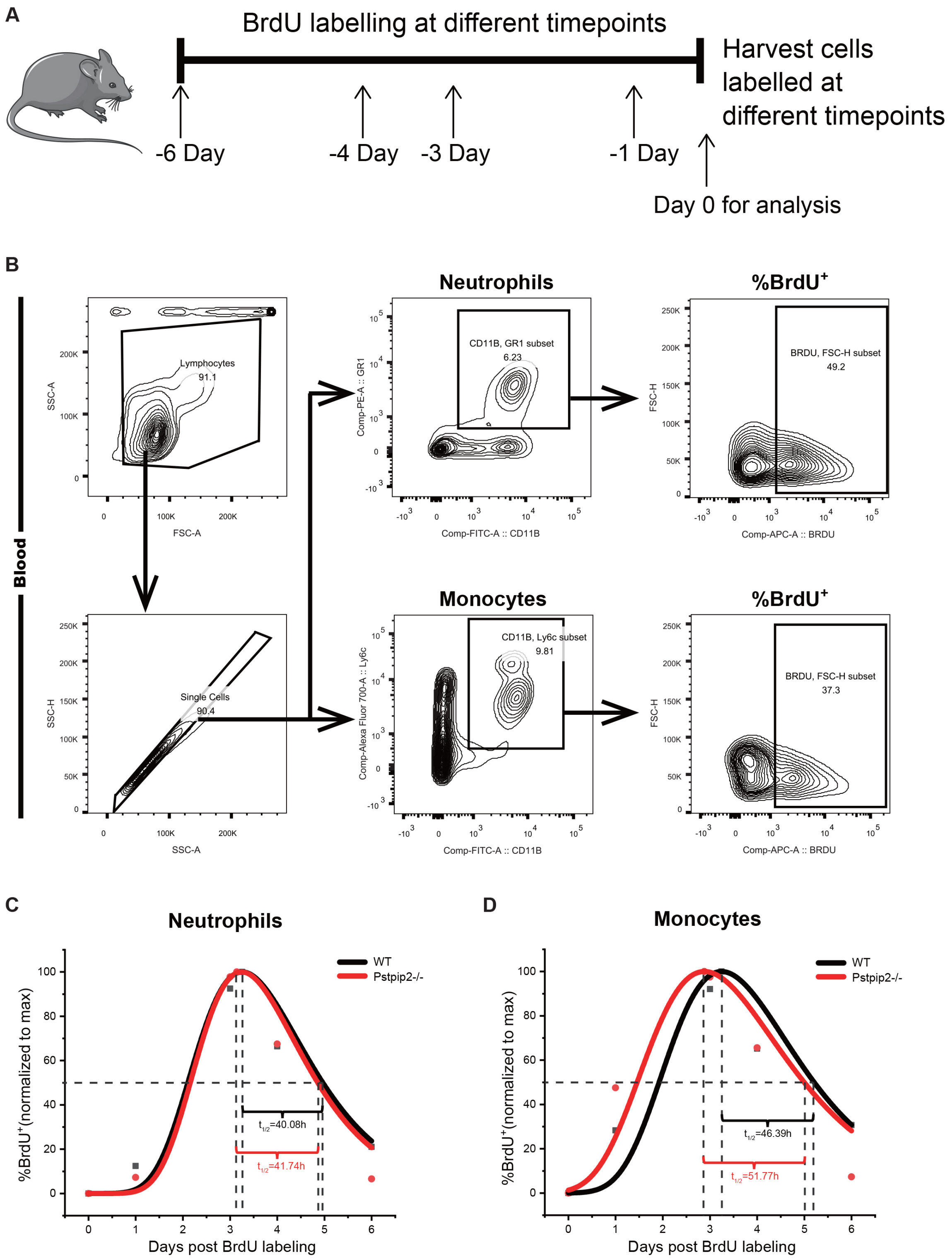

Fig. S3. Measuring the half-life time of myeloid cells by BrdU chasing.

(A) Experiment procedures.

(B) Gating strategies.

(C) Half-life time in neutrophils from WT and Pstpip2 mutant.

(D) Half-life time in monocytes from WT and Pstpip2 mutant.

Disease Models & Mechanisms • Supplementary information

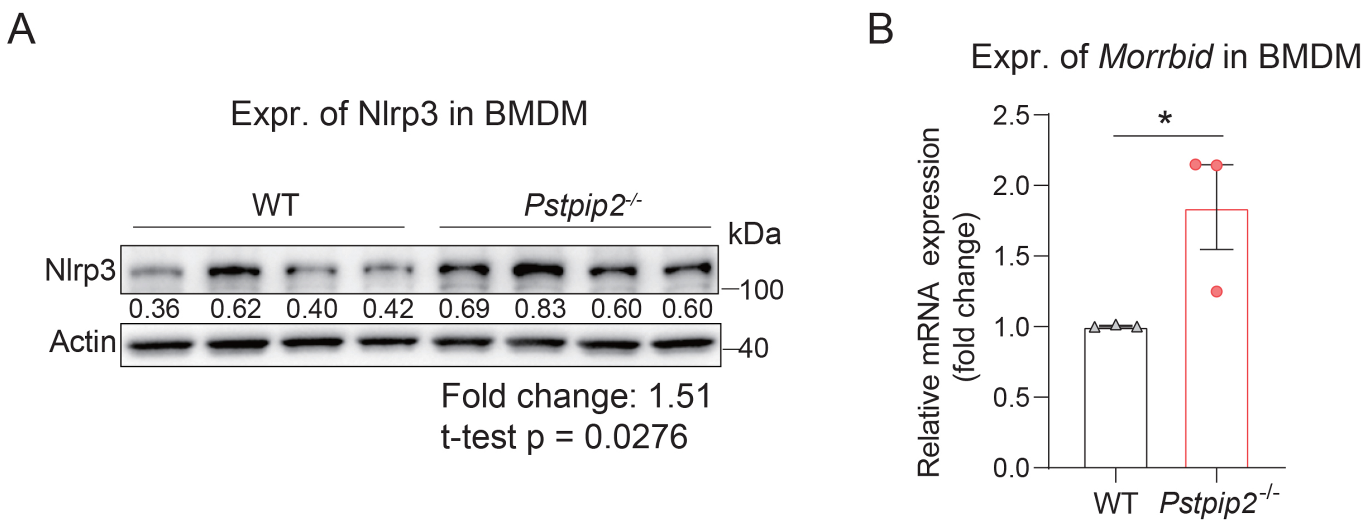

Fig. S4. Measuring inflammation activity using cellular models. BMDM cells were isolated from WT and Pstpip2 mutant for immunoblotting assays and qRT- PCR assays.
(A) Expression of Nlrp3 in BMDM.
(B) Expression of *Morrbid* in BMDM.

Disease Models & Mechanisms • Supplementary information

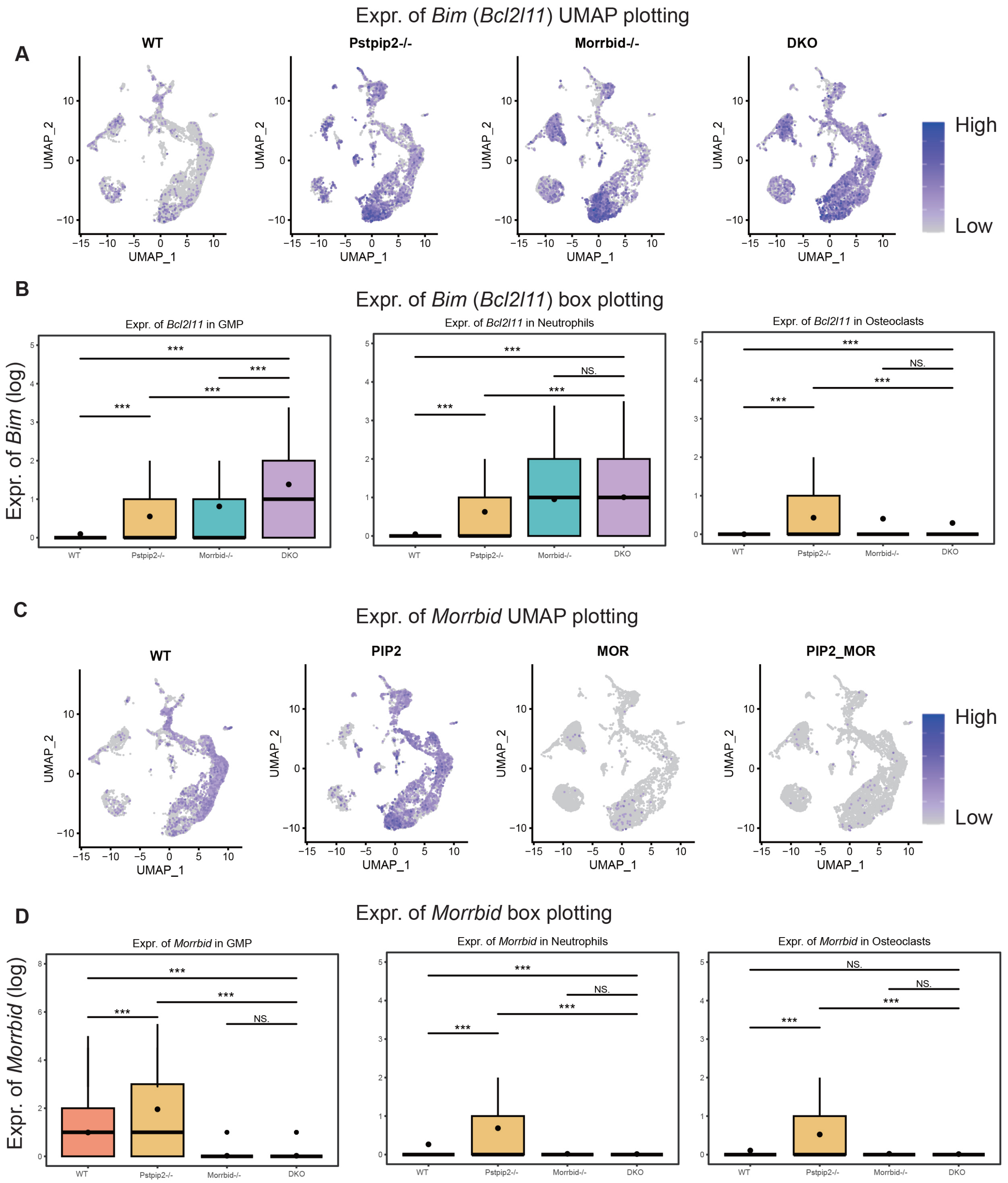

Fig. S5. Expressio of Bim and Morrbid in the scRNA-seq datasets.

(A-B) Expression of Bim in the scRNA-seq datasets, visualized by UMAP feature plotting or by box plotting for quantification.

(A-B) Expression of lncRNA *Morrbid* in the scRNA-seq datasets, visualized by UMAP feature plotting or by box plotting for quantification.

Disease Models & Mechanisms • Supplementary information

Table S1. Primer sequences used for quantitative RT-PCR are listed below:

Gene	Orientation	Sequence (5'-3')
Il1b	Forward primer	CAACCAACACGTGATATTCTCCATG
	Reverse primer	GATCCACACTCTCCAGCTGCA
Nlrp3	Forward primer	ATTACCCGCCCGAGAAAGG
	Reverse primer	TCGCAGCAAAGATCCACACAG
Mmp13	Forward primer	CTTCTTCTTGTTGAGCTGGACTC
	Reverse primer	CTGTGGAGGTCACTGTAGACT
Acp5	Forward primer	CACTCCCACCCTGAGATTTGT
	Reverse primer	CATCGTCTGCACGGTTCTG
Csf1r	Forward primer	ATGAGCAGGAGTATTGCCAAGG
	Reverse primer	TCCATTCCCAATCATGTGGCTA
Nfkb2	Forward primer	GGCCGGAAGACCTATCCTACT
	Reverse primer	CTACAGACACAGCGCACACT
Rela	Forward primer	AGGCTTCTGGGCCTTATGTG
	Reverse primer	TGCTTCTCTCGCCAGGAATAC
Prdx1	Forward primer	AATGCAAAAATTGGGTATCCTGC
	Reverse primer	CGTGGGACACACAAAAGTAAAGT
Prg4	Forward primer	GAAAATACTTCCCGTCTGCTTGT
	Reverse primer	ACTCCATGTAGTGCTGACAGTTA
Bcl6	Forward primer	TAGAGCCCATAAGACAGTGCT
	Reverse primer	CACCGCCATGATATTGCCTTC
Actb	Forward primer	CCAGAGCAAGAGAGGTATCC
	Reverse primer	CTGTGGTGGTGAAGCTGTAG

Disease Models & Mechanisms • Supplementary information
